# Thermal damage induced changes in optical properties of the porcine dermis

**DOI:** 10.1117/1.JBO.30.10.105003

**Published:** 2025-10-11

**Authors:** Anjelyka T. Fasci, Maria A. T. Hoffman, Andrea L. Smith, Matthew E. Macasadia, Amanda J. Tijerina, Robert Lyle Hood, Michael P. DeLisi, Joel N. Bixler

**Affiliations:** a SAIC, JBSA Fort Sam Houston, Texas, United States; b University of Texas at San Antonio, Department of Mechanical Engineering, San Antonio, Texas, United States; c Conceptual Mindworks, Inc., San Antonio, Texas, United States; d Texas A&M University, Department of Biomedical Engineering, College Station, Texas, United States; e Air Force Research Laboratory, JBSA Fort Sam Houston, Texas, United States

**Keywords:** skin properties, double-integrating spheres, thermal damage, tissue preparation, inverse adding-doubling method, optical properties

## Abstract

**Significance:**

Understanding thermal effects on tissue optical properties is fundamental for optimizing laser-based medical interventions. We address the critical knowledge gap of temperature-dependent changes in porcine dermis optical properties.

**Aim:**

We explore the thermal damage influence on the excised dermis optical properties at wavelengths from 400 to 1100 nm.

**Approach:**

Using a double-integrating-sphere system and inverse adding-doubling, we determined absorption, $\mu_{a}$, and reduced scattering, $\mu_{s}^{\prime }$, coefficients before and after a 2.5-min thermal exposure.

**Results:**

We observed non-linear changes in both $\mu_{a}$ and $\mu_{s}^{\prime }$ across temperature regimes. Minimal changes occurred at 37°C and 43°C. At 50°C, slight increases in both coefficients were observed. Significant alterations occurred at 60°C, with substantial increases in $\mu_{s}^{\prime }$ and variable changes in $\mu_{a}$ depending on wavelength region. At 70°C, $\mu_{s}^{\prime }$ values remained elevated, whereas $\mu_{a}$ showed mixed responses, with some wavelength regions decreasing, indicating progressive structural breakdown. The Arrhenius damage model showed an exponential increase with temperature.

**Conclusions:**

We reveal complex thermal-induced changes in tissue optical properties, particularly at higher temperatures. Findings reinforce a critical threshold between 50°C and 60°C where significant changes occur. The non-linear, wavelength-dependent responses emphasize the need for comprehensive data in laser–tissue interaction modeling, with important implications for optimizing laser-based medical treatments.

## 1 Introduction

Laser technology has improved modern medicine by enabling unprecedented precision in both diagnostic procedures and therapeutic interventions,^1^^,^^2^ transforming how clinicians approach patient care. With ongoing progress in laser technology, it is imperative to deepen our understanding of laser–tissue interactions, particularly concerning heat-induced damage. Laser-induced thermal damage to tissues is a complex process, involving coagulation and vaporization, which result in distinct layers of tissue damage.^3^ Accurately modeling these effects is necessary for predicting tissue responses to laser exposure. Although Monte Carlo simulations excel at optical modeling, they lack direct links to heat transfer and tissue damage kinetics, necessitating coupling with thermal models.^4^^–^^6^ Finite element multiphysics simulations, however, offer a more holistic approach by integrating heat transfer and damage accumulation, thus providing a comprehensive understanding of laser–tissue interactions.^7^^–^^9^

To quantify the extent of cumulative thermal damage in tissues, the Arrhenius damage model is widely employed. This model, based on the Arrhenius equation, describes the kinetics of thermal denaturation of proteins and other biomolecules. It relates the rate of damage accumulation to temperature and time, allowing prediction of tissue necrosis and coagulation.^10^^–^^14^ The Arrhenius model is particularly useful in laser–tissue interaction studies as integration with heat transfer simulations provides a more complete picture of the damage process.^12^^,^^15^^,^^16^

Several studies have contributed to our understanding of the thermal effects of laser exposure on biological tissues. Barton et al.^17^ observed that pulsed laser exposure on hamster dorsal skin flaps led to hemoglobin deoxygenation, blood coagulation, and blood vessel constriction. In addition, pulsed laser exposure contributed to tissue heating, along with a notable increase in tissue absorption coefficients. Specifically, the study reported that met-hemoglobin exhibited absorption coefficients 29 times higher than oxy-hemoglobin and 3.7 times higher than deoxy-hemoglobin at 632 nm. Laufer et al.^18^ employed an integrating sphere system and an inverse Monte Carlo method to measure temperature-induced changes in absorption and reduced scattering in *ex vivo* human dermis. They found that between 650 and 1000 nm, the transport scattering coefficient increased by 0.47% per degree Celsius for dermis while decreasing by 0.14% per degree Celsius for the subdermis as tissue temperature rose from 25°C to 40°C, with scattering coefficients ranging from 1 to $75\;{\mathrm{mm}}^{-1}$. In addition, Iorizzo et al.^19^ investigated scattering coefficients at various wavelengths (400 to 1650 nm) and temperatures (25°C, 36°C, and 60°C) using *in vivo* mouse ear skin tissue. They observed scattering coefficients between 4 and $8\;{\mathrm{mm}}^{-1}$, with values decreasing with increasing wavelength, particularly pronounced at 60°C compared with lower temperatures. The absorption coefficients, on the other hand, showed an opposite trend of increasing with temperature, albeit fading at 1300 nm. These collective findings showcase the complex connection between laser-induced thermal effects and tissue optical properties and emphasize the importance of further research in this domain.

Jaunich et al.^20^ developed a computational model for analyzing temperature distributions and heat-affected zones in skin tissue irradiated by short-pulse lasers. Their experiments on multi-layer tissue phantoms and mouse skin samples provided insights into biological tissue thermal response. Using a Q-switched pulsed 1064 nm Nd:YAG laser (200-ns pulse width) and a 1552-nm diode short-pulsed laser (1.3-ps pulse width), they measured axial and radial temperature distributions, comparing them with numerical models. Their murine tissue measurements provided optical property data for three skin layers at wavelengths of 1064 and 1552 nm, with absorption coefficients ranging from 0.04 to $1\;{\mathrm{mm}}^{-1}$ and scattering coefficients between 7 and $12.5\;{\mathrm{mm}}^{-1}$ depending on the layer and wavelength. DeLisi et al.^16^ developed a computational model focusing on near infrared (NIR) laser photothermal response in lightly pigmented skin. Their study demonstrated accurate temperature history matches with experimental data across a broad exposure time range, highlighting the significance of incorporating NIR optical properties consistent with the expected distribution of chromophores in skin tissue, particularly melanin. By deriving values for epidermal optical absorption coefficients ($0.35\;{\mathrm{mm}}^{-1}$ for the epidermis and $0.17\;{\mathrm{mm}}^{-1}$ for the dermis) and integrating other relevant material properties, their simulations produced temperature profiles in alignment with experimental thermography.

Our investigation examines how thermal damage affects tissue optical properties. These properties are significant in determining how tissue responds to laser exposure.^21^^–^^23^ This research aims to deepen our understanding of how laser-induced thermal damage develops in tissue.^5^^,^^8^^,^^9^^,^^24^^,^^25^ Our findings have direct applications in laser-induced thermal therapy, where precise control of tissue damage is essential for minimally invasive treatments. This work both builds on existing research and addresses current knowledge gaps by characterizing the temperature-dependent changes in tissue optical properties, providing essential experimental data that may inform future thermal modeling and potentially contribute to improved precision in laser-based medical treatments.

## 2 Methods

### 2.1 Sample Collection and Preparation

The experimental protocols utilized skin tissue obtained from the flanks of a female Yucatan miniature pig (age: 3 to 6 months) immediately following euthanasia. An Institutional Animal Care and Use Committee (IACUC)-approved tissue-sharing arrangement protocol enabled post-mortem collection from animals involved in separate authorized research. The animals were procured, maintained, and used according to an IACUC-approved Animal Use Protocol and established animal welfare standards, compliant with the following: DoD Instruction 3216.01 (DoD, 2019); U.S. Department of Agriculture Animal Welfare Regulations (USDA, 2013); the Guide for the Care and Use of Laboratory Animals, 8th Edition, National Research Council (2011); and AFMAN 40-401(1) (U.S. Air Force, 2005). The Air Force Research Laboratory at Joint Base San Antonio Fort Sam Houston, Texas, has been accredited by AAALAC International since 1967. Bulk sections of tissue were placed in airtight plastic bags shortly after collection and maintained in a −20°C laboratory freezer until use in the present study. This specific animal model was selected due to the well-documented similarities between Yucatan miniature pig skin and human skin in terms of optical, physiological, and structural characteristics within this age range.^26^^,^^27^ Of particular relevance is the melanin content and distribution pattern in the Yucatan mini-pig epidermis, which more closely approximates human skin compared with less pigmented breeds such as Yorkshire pigs.^28^ Although epidermal tissue was not included in the current investigation, the choice of this established human skin analog enhances the applicability of these findings to laser exposure research.

**Fig. 1:**
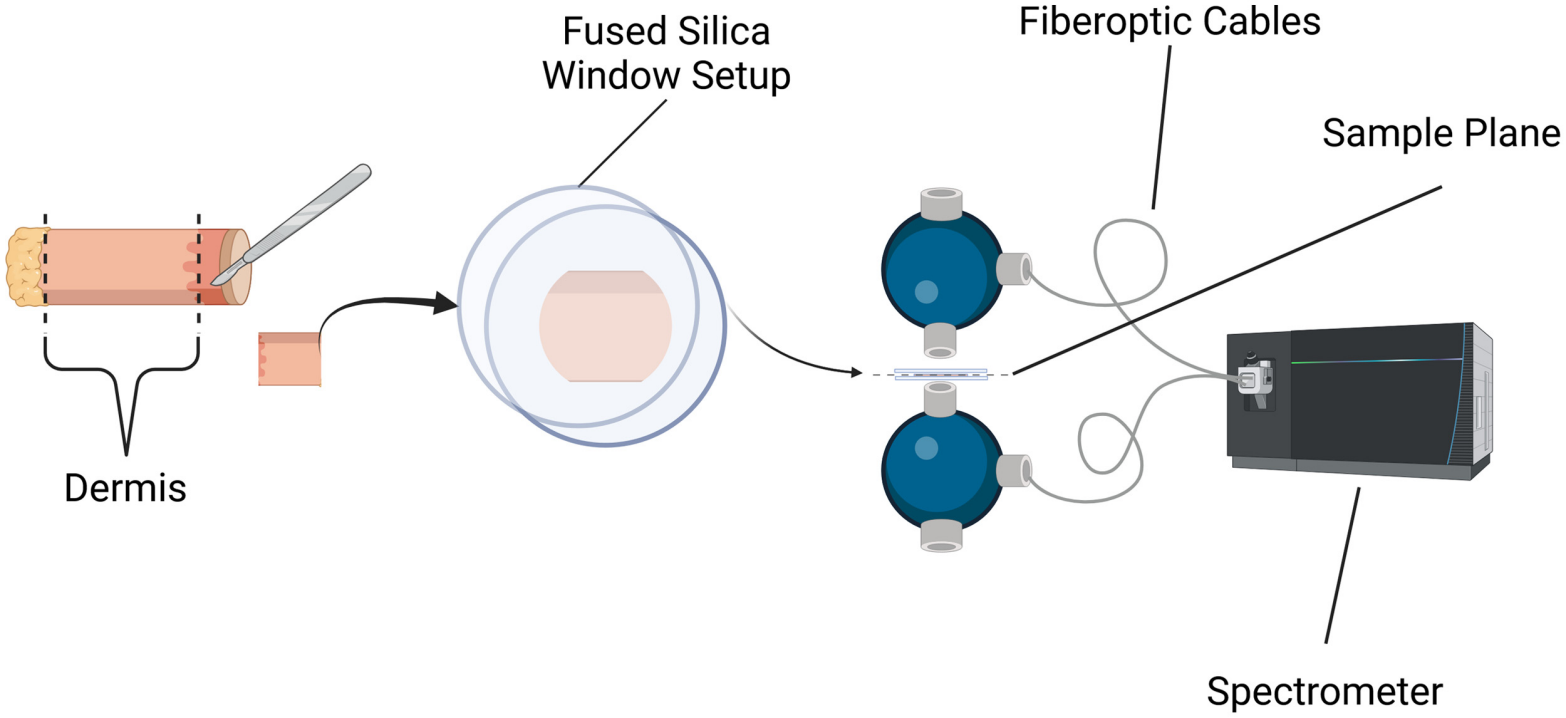
Sample collection and mounting. The system consists of three main components: a dermis sample preparation station, a sample mounting setup, and a spectroscopic measurement apparatus. First, the dermis tissue is precisely sectioned. The sectioned sample is mounted between two fused silica windows. The mounted sample forms the sample plane, which is positioned between dual integrating spheres connected by fiberoptic cables. Created in BioRender. Fasci, A. (2025).^29^

Porcine dermis samples were obtained by coring 10-mm-diameter columns from the frozen flanks of pig specimens, ensuring consistency in the size and location of the samples. The dermis was manually sectioned using a dissection microscope and razor blade to achieve 1.19±0.29  mm thickness for each sample (Fig. 1). These samples were wrapped in saline-moistened gauze and stored in press-seal bags with excess air expelled. They were then placed in a 4°C refrigerator until it was time to conduct optical property measurements, typically ranging from 1 to 5 h.

To maintain the physiological conditions of the porcine dermis, we adopted a standardized approach for sample treatment. All dermis samples underwent immersion in a 0.9% normal saline solution (NSS) for a maximum of 15 min. This process allowed the minimization of variations in hydration levels across different samples and ensured the optical properties were primarily influenced by the inherent characteristics of the tissue.

### 2.2 Optical Setup and Measurements

**Fig. 2:**
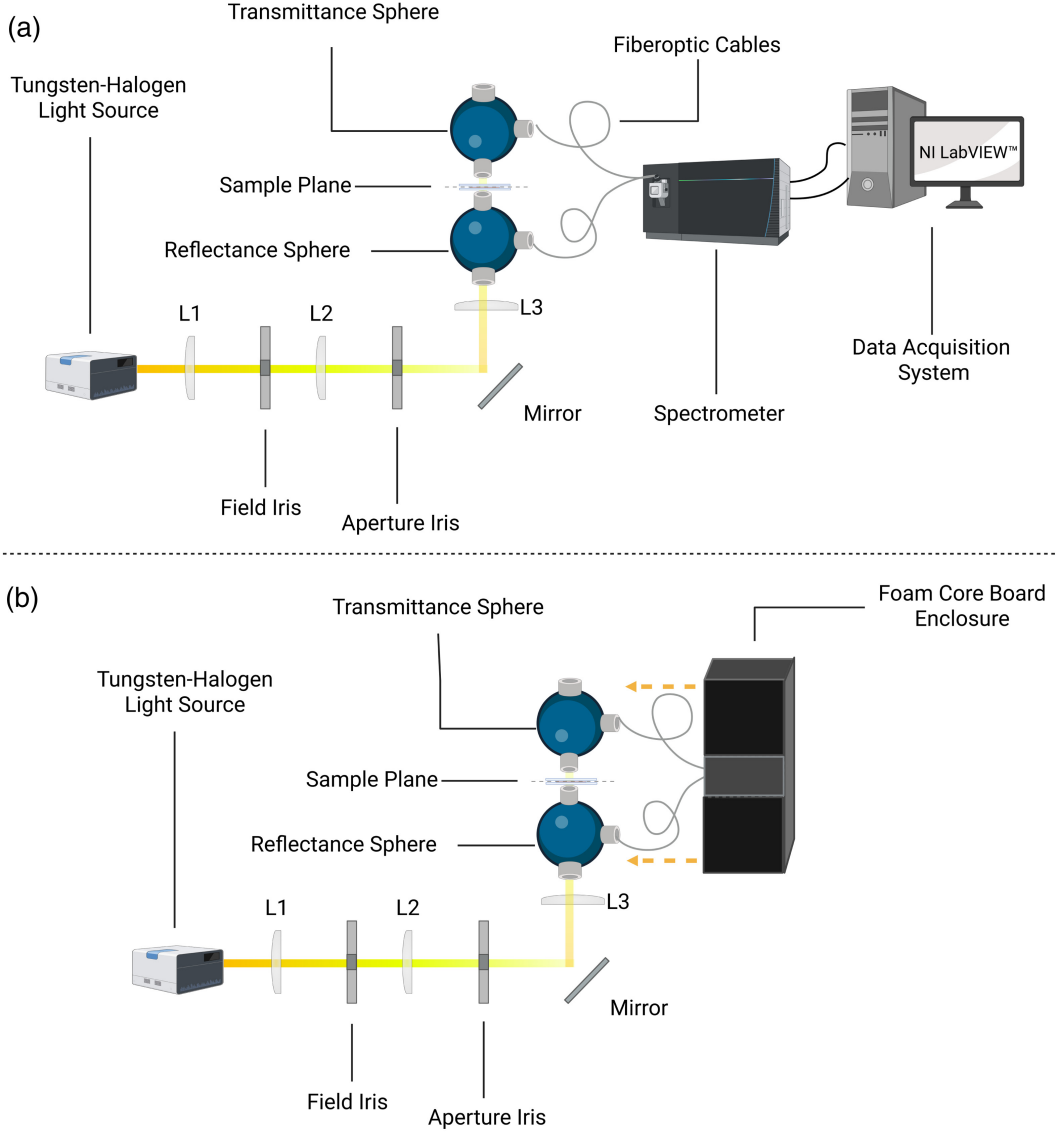
Comprehensive optical property measurement system diagram. (a) Standard configuration showing the dual integrating sphere setup with tungsten–halogen light source, optical components (lenses L1, L2, and L3; field iris; aperture iris; and mirror), and data acquisition system. (b) Modified configuration with foam core board enclosure (shown in black) surrounding the integrating spheres to minimize external interference. Each integrating sphere is equipped with Spectraflect^®^-coated port plugs and couples to 600-μm-core, 0.22 NA optical fibers via subminiature version A (SMA) port caps. The integrated reflection and transmission spectra are collected by a dual-channel spectrometer featuring a 500-nm blaze grating and a 50-μm slit, enabling high-precision optical characterization of the tissue samples. Created in BioRender. Fasci, A. (2025).^31^

The optical property measurement method is identical to Hoffman et al.^30^ In summary, the optical characterization utilized a single-beam dual integrating sphere system (Fig. 2) consisting of two 3.3″ Spectralon integrating spheres (4P-GPS-033-SL, Labsphere, North Sutton, New Hampshire) with specific port configurations (1.5″ sample port and 1″ auxiliary ports). Illumination was provided by a tungsten halogen source (66184, Oriel) and controlled through a lens system comprising a condenser lens (L1, 38-mm focal length) and two plano-convex lenses (L2 and L3, 150-mm focal length) in a 4f configuration, delivering a uniform 1-mm-diameter spot to the sample. Dermis specimens were mounted between fused silica windows (Edmund Optics #34-599, Barrington, New Jersey, United States) for both thickness determination and optical measurements. The ∼10-mm-diameter dermis samples were sufficiently large to completely cover the 6.35-mm integrating sphere port, minimizing light loss that could compromise inverse adding doubling (IAD) measurements. Tissue thickness was measured using a dial indicator (GA-725, Vigor, Portland, Oregon, United States) with thousandths of a millimeter precision on the mounted sample assembly. The dial gauge was positioned to contact the glass surface with minimal pressure to avoid tissue compression that could affect thickness accuracy. The thickness measurement protocol involved documenting tissue thickness in a spreadsheet that was coded to automatically subtract each silica window thickness (1.0533 mm) to provide accurate sample thickness values for the IAD algorithm. This coverslip thickness value was the experimentally measured average thickness for n=30 glass windows with a standard deviation of 0.028 mm. This approach ensured that thickness measurements were performed on the same mounted configuration that was then used for optical property measurements. Thickness measurements of each tissue sample are provided as Table S1 within the Supplementary Material. Prior to conducting tissue optical property measurements, system calibration used a reference polyurethane phantom (BioPixS0064, BioPixS Ltd., Cork, Ireland) to establish baseline performance. Additional details regarding the phantom specifications and calibration measurements are available in the Supplementary Material. Figure S1 provides violin plots of the BioPix phantom measurements for three wavelengths.

A custom enclosure using black foam-core board [shown in Fig. 2(b)] was implemented to minimize external interference during optical measurements. The enclosure was specifically designed to enclose both integrating spheres while maintaining space for fiber-optic cables and ensuring unobstructed operation of the mirror and lens assembly (L1, L2, and L3). This protective housing effectively blocked ambient light sources and external disturbances, ensuring reliable and reproducible experimental results throughout the measurement process.

### 2.3 Thermal Treatment Protocol

**Fig. 3:**
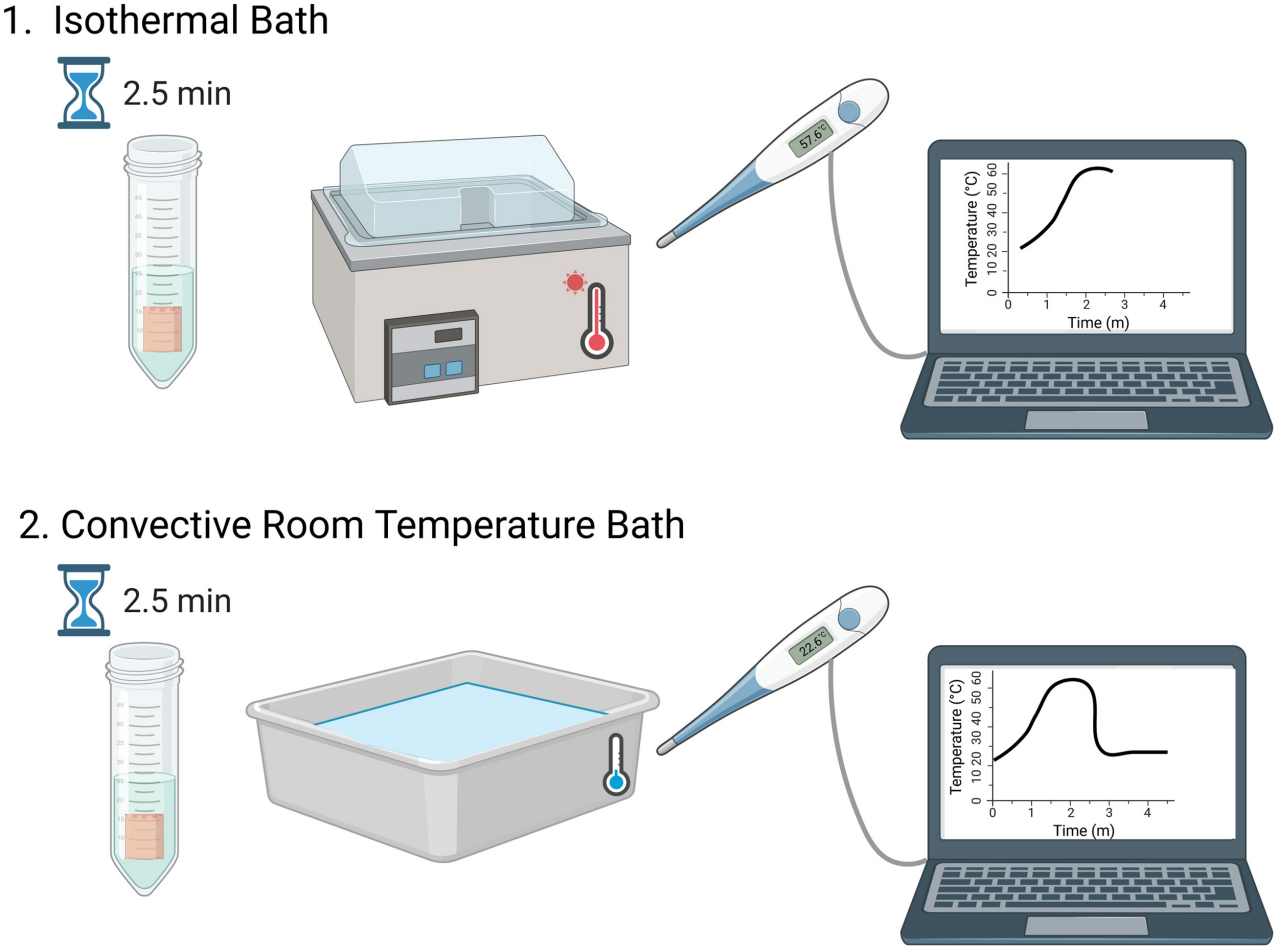
Schematic representation of the thermal treatment protocol. (1) Tissue samples are submerged in an isothermal water bath at the target temperature for 2.5 min, followed by (2) transfer to a room-temperature water bath for 2.5 min of cooling. Environmental temperature is continuously monitored using a thermistor placed in a separate test tube containing 3 mL of NSS. Representative temperature–time traces are shown for each phase, demonstrating the heating and cooling profiles throughout the thermal treatment process. Created in BioRender. Fasci, A. (2025).^32^

A thermal treatment protocol (Fig. 3) was implemented following baseline pre-exposure optical property measurements to investigate temperature-dependent changes in porcine dermis samples. The samples were carefully transferred from custom sample holders to test tubes (Pyrex^®^ flat-bottom culture tubes, rimless) containing 3 mL of NSS, with their orientation maintained. Temperature monitoring was achieved using two calibrated thermistors (EPCOS - TDK Electronics, B57540G1103F000, Munich, Germany). One thermistor was placed in a test tube containing 3 mL of NSS and was used to monitor the environmental bath temperature. The second thermistor was used for monitoring the internal temperature of a representative tissue sample. A dermis specimen from the same experimental collection group was punctured using a Monoject™ Needle (20  g×1  inch), creating a partial-thickness insertion point that did not penetrate completely through the tissue. The thermistor was then gently inserted into this puncture site, allowing it to remain embedded within the tissue rather than exiting through the opposite side. This instrumented tissue sample was placed in its own test tube containing 3 mL of NSS to maintain consistent experimental conditions and was not used for any optical property measurements.

The tissue samples, contained within their test tubes, were subjected to thermal treatment in a temperature-controlled water bath filled with ∼18  L of distilled water (Stuart SBS40 heated water bath, United States, 24 L capacity) at selected temperatures: room temperature (∼22°C), 37°C, 43°C, 50°C, 60°C, and 70°C. The selection of these temperatures was strategically designed to span physiologically relevant conditions (37°C), the established damage threshold (43°C being the highest temperature at which no damage was observed, regardless of exposure duration, with 44°C showing damage after 420 min of exposure),^10^ and higher temperatures to investigate more severe thermal effects on dermal tissue structure and properties. The 50°C condition was included to examine rapid thermal damage, as previous studies demonstrated tissue damage occurring between 2 and 4 min of exposure at this temperature.^10^^,^^11^^,^^13^^,^^33^ The room temperature group served as the control.

Each thermal treatment consisted of a 2.5-min exposure at the target temperature, followed by a 2.5-min cooling period. All test tubes were positioned in a test tube rack and simultaneously placed into the heated water bath for the exposure phase. After 2.5 min, the entire rack was removed and immediately transferred to a separate room-temperature water bath containing ∼2  L of distilled water for the cooling phase. A calibrated thermometer placed in the cooling bath confirmed the room temperature environment (∼22°C). After the 2.5-min cooling period, the test tube rack was removed from the cooling bath. Following the thermal protocol, samples were carefully removed from their test tubes and positioned among glass slides, maintaining their original orientation from the baseline measurements. Sample thicknesses were remeasured to account for any thermal-induced dimensional changes. Optical property measurements were then repeated to quantify temperature-induced alterations in the tissue’s reflectance and transmittance characteristics.

### 2.4 Data Processing

**Fig. 4:**
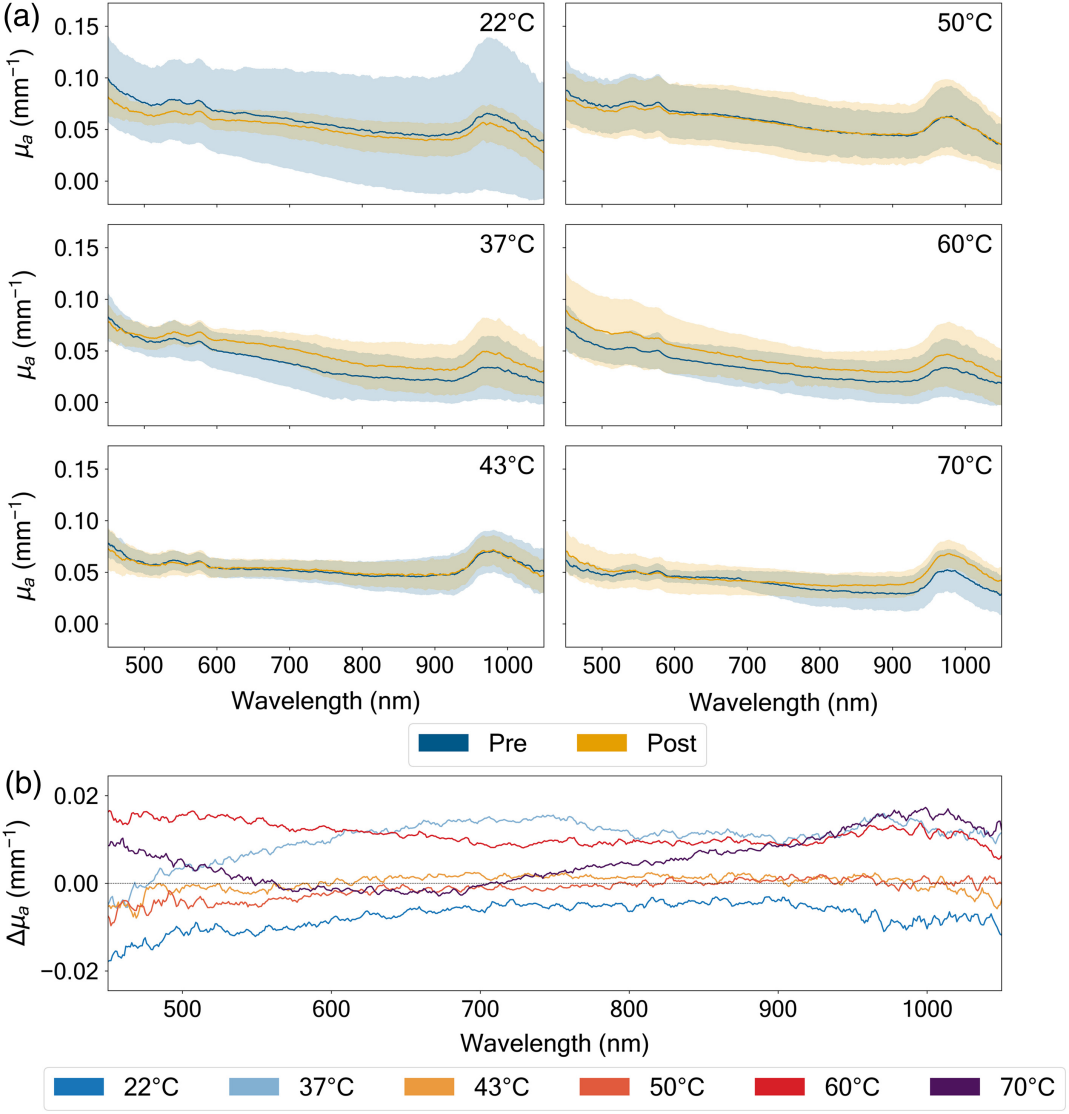
Pre- versus post-thermal treatment of absorption coefficient ($\mu_{a}\;[{\mathrm{mm}}^{-1}]$) spectra of the porcine dermis measured across wavelengths (450 to 1050 nm) at six distinct temperatures (22°C, 37°C, 43°C, 50°C, 60°C, and 70°C). The thermal treatment duration was 2.5 min. (a) Absolute $\mu_{a}$ values, with pre-treatment measurements shown in blue and post-treatment in yellow, accompanied by their respective standard deviations (shaded regions, all values reported as mean ± SD (n=7) after outlier removal using z-score analysis (Sec. 2.4). (b) Absolute difference ($\Delta \mu_{a}$) between pre- and post-treatment conditions, with distinct colored traces corresponding to measurements at each temperature. The spectral features reveal characteristic hemoglobin absorption bands at shorter wavelengths (500 to 600 nm) and water absorption near 970 nm. Temperature-dependent changes in $\Delta \mu_{a}$ show varying patterns, with 37°C exhibiting increasing positive differences at longer wavelengths, whereas 60°C shows positive differences throughout the spectrum (+29.6% for water and +26.7% for hemoglobin), indicating increased absorption post-treatment.

The details of the data processing procedure were reported previously in Hoffman et al.^30^ The specific measurement protocol used here follows the methodology outlined in Fig. 4 of Prahl’s IAD manual for dual sphere $M_{R}$ and $M_{T}$ measurements.^34^

The dual sphere approach requires multiple measurement configurations: sample measurements with both spheres active, standard reflectance calibration, transmittance calibration with open port, and background measurements with blocked beam, which differs slightly from the single sphere measurements. In our experimental implementation, normalized transmittance T(λ) was calculated using

$$T(\lambda )=\frac{S_{t}(\lambda )-N_{t}(\lambda )}{F_{t}(\lambda )-N_{t}(\lambda )},$$ (1)

where the measurement terms correspond to the specific configurations: $S_{t}(\lambda )$ represents the sample’s transmission measurement, $N_{t}(\lambda )$ denotes null/background signals with blocked beam, and $F_{t}(\lambda )$ indicates the transmittance sphere’s full throughput response with open port.

The normalized reflectance coefficient R(λ) was calculated as

$$R(\lambda )=\rho (\lambda )\cdot \frac{S_{r}(\lambda )-N_{r}(\lambda )}{P_{r}(\lambda )-N_{r}(\lambda )},$$ (2)

where ρ(λ) represents the calibration standard’s reflectance, $S_{r}(\lambda )$ indicates the sample’s reflection measurement, $N_{r}(\lambda )$ denotes the background reflection signal, and $P_{r}(\lambda )$ represents the calibration standard’s reflection measurement.

These experimental measurements correspond to the theoretical IAD framework for a dual-sphere configuration, where the measured total reflectance and transmittance values are processed through the IAD algorithm to extract the fundamental optical properties. Data processing was performed using IAD version 3.16.3 (released May 2024) with an anisotropy factor of g=0.9 applied to account for the typical forward-scattering behavior of biological tissues. A recently calibrated 40% reflection standard (FSS-08-01c, Avian Technologies, New London, New Hampshire, United States) was used in combination with a Spectraflect-coated port reducer to calculate the normalized reflectance.

To ensure data quality and group consistency, outlier identification and removal were performed using sample-level z-score analysis. For each temperature group (n=10−15), the mean absorption coefficient ($\mu_{a}$) and reduced scattering coefficient ($\mu_{s}^{\prime }$) values were calculated across all wavelengths for each individual sample, followed by z-score computation of these sample means within each group. Samples with combined z-scores exceeding acceptable thresholds were removed through an iterative optimization process that minimized outlier influence while maintaining consistent sample sizes across groups (n=7 per group). This approach removed samples typically exhibiting z-scores greater than ±1.5 for either $\mu_{a}$ or $\mu_{s}^{\prime }$ values.

### 2.5 Arrhenius Damage Model and Thermal Injury Assessment

The Arrhenius damage model is a widely accepted approach in thermal biology for quantifying tissue damage as a function of temperature and exposure time.^35^^,^^36^ This model, based on the Arrhenius equation, has been successfully applied to predict irreversible thermal alterations in structural proteins and tissues.^37^ Our analysis of thermal damage in porcine dermis samples employed this model, which is expressed as Eq. (3)

$$\Omega (t)=A\int_{0}^{t}\exp(-\frac{E_{a}}{RT(\tau )})d\tau ,$$ (3)

where Ω(t) is the damage parameter, A is the frequency factor ($s^{-1}$), $E_{a}$ is the activation energy ($J\cdot {\mathrm{mol}}^{-1}$), R is the universal gas constant ($8.314\;J\cdot {\mathrm{mol}}^{-1}\cdot K^{-1}$), T(τ) is the absolute temperature of the tissue as a function of time τ (K), and t is the total heating time (s).^10^

The Arrhenius model assumes thermal damage follows first-order reaction kinetics, where the rate of damage is proportional to the concentration of undamaged tissue.^10^ For our analysis, we used the temperature–time profiles recorded during the thermal treatment protocol (Sec. 2.3). The values for A ($2.74\times 10^{94}$) and $E_{a}$ ($5.90\times 10^{5}$) were obtained from the literature specific to porcine skin.^16^ We calculated Ω(t) for each temperature condition, considering both the 2.5-min heating phase and the 2.5-min cooling phase.

The degree of thermal damage was assessed based on the following criteria:^33^

• Ω<0.53: reversible damage
• 0.53≤Ω<1: irreversible damage threshold
• Ω≥1: complete thermal necrosis.

These calculated damage parameters were then correlated with the observed changes in optical properties ($\mu_{a}$ and $\mu_{s}^{\prime }$) obtained from the IAD method (Sec. 2.4). This established relationships between the extent of thermal damage and alterations in tissue optical characteristics across different temperature conditions.

## 3 Results and Discussion

Thermal treatment of porcine dermis induced variable changes in optical properties across temperatures ranging from 22°C to 70°C. Analysis revealed distinct patterns in both absorption coefficient ($\mu_{a}$) and reduced scattering coefficient ($\mu_{s}^{\prime }$) modifications, with temperature-dependent effects observed across the measured wavelength range (450 to 1050 nm).

### 3.1 Analysis of Optical Property Changes Per Thermal Exposure Group

The thermal treatment induced distinct changes in tissue absorption properties (Fig. 4), with the magnitude and direction of these changes varying significantly across both temperature and wavelength. In the physiological temperature range (37°C to 43°C), the tissue demonstrated relatively stable optical absorption characteristics, with only subtle modifications to the absorption coefficient that remained within $\pm 0.01\;{\mathrm{mm}}^{-1}$. This stability suggests moderate heating preserves the fundamental absorption properties of the tissue’s chromophores.

The most pronounced spectral alterations in absorption occurred at temperature extremes, particularly in the near-infrared region (900 to 1000 nm). At room temperature (22°C), we observed a decrease in absorption in the hemoglobin region ($\Delta \mu_{a}=-13.9\pm 12.2\%$), though the high standard deviation suggests this effect may not be statistically significant. The water-dominant region showed a larger decrease ($\Delta \mu_{a}=-20.9\pm 57.9\%$). Conversely, exposure to higher temperatures (60°C to 70°C) resulted in variable changes in the hemoglobin region ($\Delta \mu_{a}=+26.7\pm 56.9\%$ and −0.48±29.3%), potentially reflecting thermal denaturation of key absorbing molecules. These bidirectional changes indicate complex underlying mechanisms in tissue response to thermal stress, with important implications for optical imaging and therapeutic applications that rely on specific spectral windows.

**Fig. 5:**
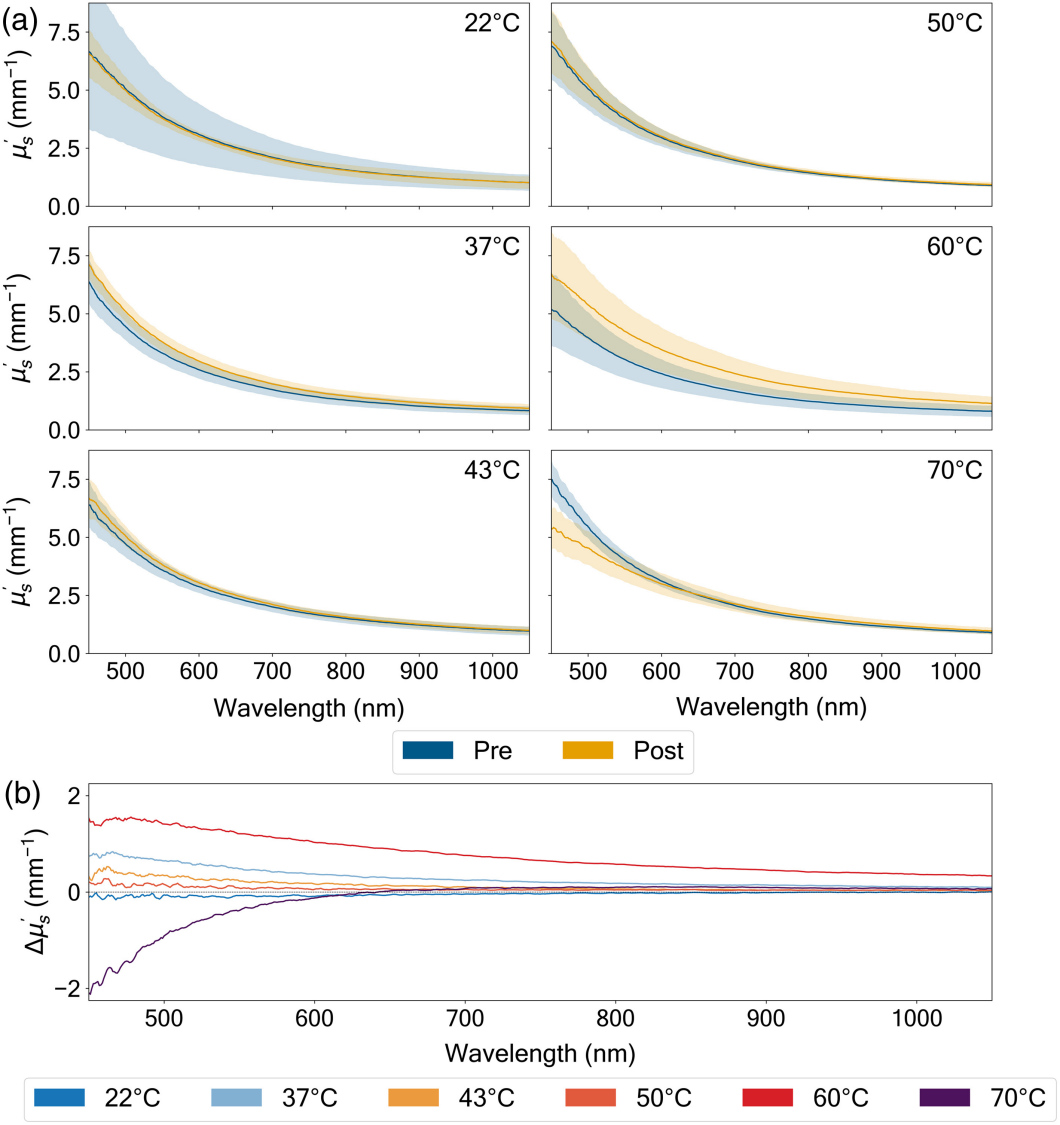
Pre- versus post-thermal treatment reduced scattering coefficient ($\mu_{s}^{\prime }\;[{\mathrm{mm}}^{-1}]$) spectra of the porcine dermis measured across wavelengths (450 to 1050 nm) at six distinct temperatures at 2.5 min each (22°C, 37°C, 43°C, 50°C, 60°C, and 70°C). (a) Absolute $\mu_{s}^{\prime }$ values, with pre-treatment measurements shown in blue and post-treatment in yellow, accompanied by their respective standard deviations [shaded regions, all values reported as mean ± SD (n=7) after outlier removal using z-score analysis, (Sec. 2.4)]. (b) Absolute difference ($\Delta \mu_{s}^{\prime }$) between pre- and post-treatment conditions, with distinct colored traces corresponding to measurements at each temperature. Notable temperature-dependent changes include the significant increase in scattering at 60°C throughout the visible spectrum, followed by a reversal at 70°C, where shorter wavelengths exhibit decreased scattering (negative $\Delta \mu_{s}^{\prime }$), transitioning to increased scattering at longer wavelengths.

The reduced scattering coefficient ($\mu_{s}^{\prime }$) demonstrated even more dramatic responses to thermal treatment (Fig. 5). Across all temperature conditions, we observed a characteristic wavelength-dependent scattering profile, with enhanced scattering at shorter wavelengths (450 to 600 nm). This wavelength dependence follows typical Mie scattering behavior, suggesting fundamental light–tissue interaction mechanisms remain intact despite thermal intervention. The data presented in Figs. 4 and 5 is also presented in Table S2 and S3 in the Supplementary Material for easy reference.

The most significant alterations in scattering properties emerged at temperatures associated with protein denaturation. Treatment at 60°C induced a substantial increase in scattering in both spectral regions ($\Delta \mu_{s}^{\prime }=+40.9\pm 36.7\%$ in the hemoglobin region and +41.9±35.9% in the water region), with the mean $\mu_{s}^{\prime }$ reaching $4.09\pm 1.07\;{\mathrm{mm}}^{-1}$ in the hemoglobin region. This marked enhancement likely reflects the initial stages of protein denaturation, where partial unfolding and aggregation create additional scattering centers.^19^^,^^38^ Notably, exposure to 70°C produced a different response in the hemoglobin region ($\Delta \mu_{s}^{\prime }=-8.03\pm 13.6\%$), whereas the water region showed a slight positive change (+6.73±17.3%). This suggests a transition to more complete protein denaturation and tissue structure breakdown, with wavelength-dependent effects.^38^^–^^42^

Even at physiological temperatures, we observed notable changes in scattering properties. Moderate heating at 37°C and 43°C resulted in increased scattering in the hemoglobin region ($\Delta \mu_{s}^{\prime }=+14.6\pm 12.8\%$ and +6.35±7.58%, respectively), indicating that even mild thermal exposure can induce measurable alterations in tissue ultrastructure. These changes, although less dramatic than those at higher temperatures, have important implications for therapeutic procedures operating in the physiological range.

### 3.2 Optical Properties in Relation to Arrhenius Damage

**Fig. 6:**
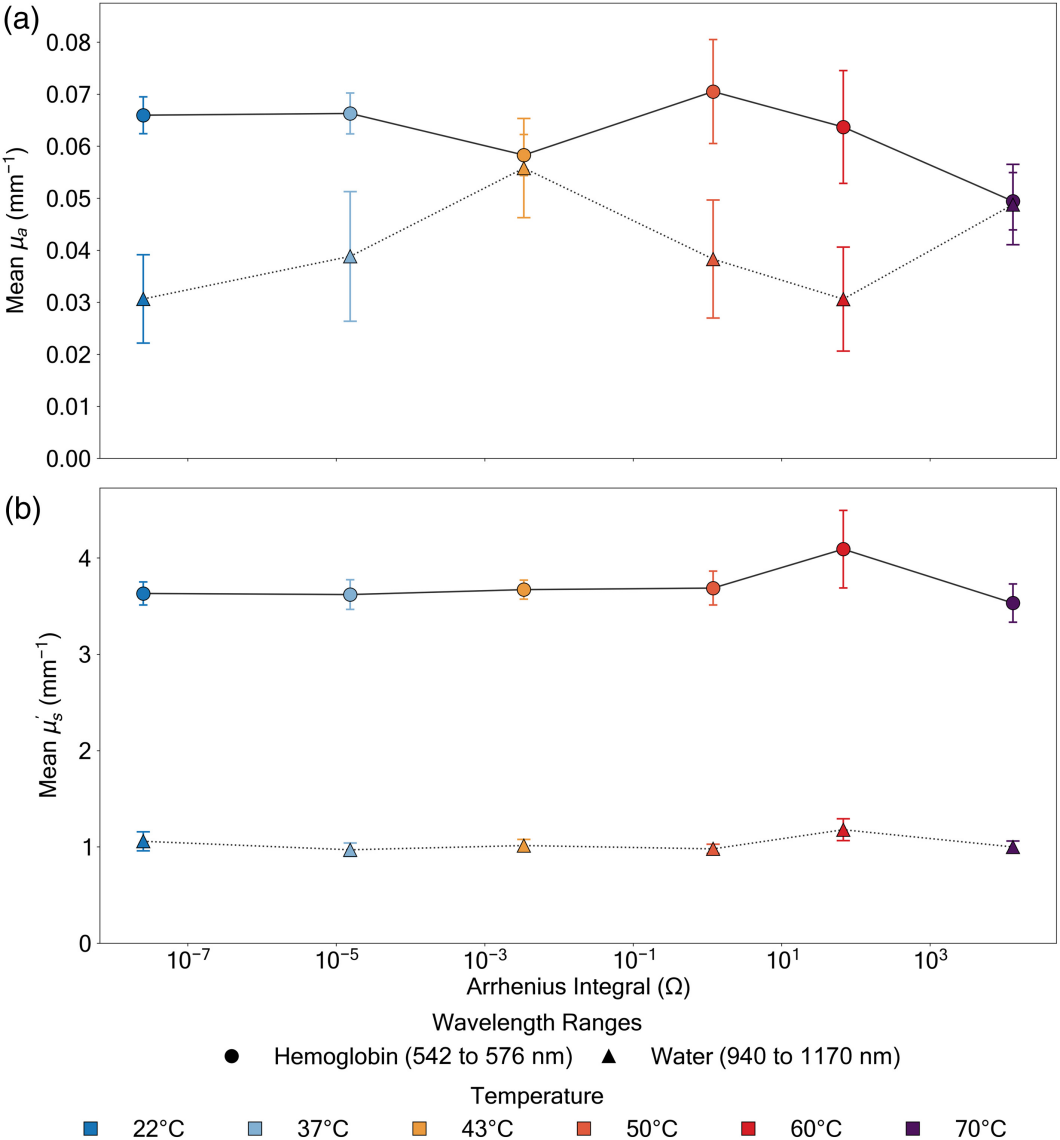
Mean absorption coefficient ($\mu_{a}$) (a) and reduced scattering coefficient ($\mu_{s}^{\prime }$) (b) of porcine dermis as a function of the Arrhenius integral (Ω) at various temperatures. Solid lines with circles represent measurements in the hemoglobin-dominant wavelength range (542 to 576 nm), whereas dotted lines with triangles represent the water-dominant wavelength range (940 to 1170 nm). All values reported as mean ± SD (n=7) after outlier removal using z-score analysis (Sec. 2.4). At room temperature (22°C, $\Omega =2.52\times 10^{-8}$), $\mu_{a}$ values were 0.066±0.009 and $0.031\pm 0.022\;{\mathrm{mm}}^{-1}$ for hemoglobin and water regions, respectively, whereas $\mu_{s}^{\prime }$ values were 3.63±0.314 and $1.06\pm 0.261\;{\mathrm{mm}}^{-1}$. Note the distinct non-monotonic pattern in scattering, particularly at 60°C ($\Omega =6.77\times 10^{1}$) where $\mu_{s}^{\prime }$ reaches $4.09\pm 1.07\;{\mathrm{mm}}^{-1}$ in the hemoglobin region.

Analysis of optical properties in relation to thermal damage revealed complex, temperature-dependent responses (Fig. 6). The absorption coefficient ($\mu_{a}$) demonstrated a non-linear behavior with the Arrhenius integral. Starting from $0.066\pm 0.009\;{\mathrm{mm}}^{-1}$ at 22°C, $\mu_{a}$ remained relatively stable at $0.066\pm 0.010\;{\mathrm{mm}}^{-1}$ at 37°C—a critical temperature associated with mild hyperthermia—before declining at higher temperatures to reach $0.064\pm 0.029\;{\mathrm{mm}}^{-1}$ at 60 °C and ultimately declining to $0.049\pm 0.015\;{\mathrm{mm}}^{-1}$ at 70°C.

The reduced scattering coefficient ($\mu_{s}^{\prime }$) showed a complex progression of structural modifications in relation to thermal damage. Beginning at room temperature ($\mu_{s}^{\prime }=3.63\pm 0.314\;{\mathrm{mm}}^{-1}$), the scattering coefficient remained stable through the physiological range to $3.62\pm 0.406\;{\mathrm{mm}}^{-1}$ at 37°C, before exhibiting a marked transition in behavior at protein denaturation temperatures (Table 1).^19^^,^^38^^–^^42^

**Table 1:** Changes in optical properties of porcine dermis at various temperatures for different wavelength ranges.

		Percent difference (%)		Post-treatment values (${\mathrm{mm}}^{-1}$)	
Temperature (°C)	Arrhenius Ω	$\mu_{a}$	$\mu_{s}^{\prime }$	$\mu_{a}$	$\mu_{s}^{\prime }$
Hemoglobin-dominant region (542 to 576 nm)					
22	$2.52\times 10^{-8}$	−13.9±12.2	−2.30±8.44	0.066±0.009	3.63±0.314
37	$1.55\times 10^{-5}$	13.0±17.7	14.6±12.8	0.066±0.010	3.62±0.406
43	$3.38\times 10^{-3}$	−2.44±17.5	6.35±7.58	0.058±0.010	3.67±0.262
50	1.19	−5.59±35.3	2.46±12.9	0.071±0.026	3.69±0.466
60	$6.77\times 10^{1}$	26.7±56.9	40.9±36.7	0.064±0.029	4.09±1.07
70	$1.31\times 10^{4}$	−0.48±29.3	−8.03±13.6	0.049±0.015	3.53±0.525
Water-dominant region (940 to 1170 nm)					
22	$2.52\times 10^{-8}$	−20.9±57.9	0.99±24.9	0.031±0.022	1.06±0.261
37	$1.55\times 10^{-5}$	4.77±88.9	8.72±20.8	0.039±0.033	0.970±0.185
43	$3.38\times 10^{-3}$	−24.4±34.2	5.04±17.4	0.056±0.025	1.01±0.168
50	1.19	2.24±80.1	5.18±13.4	0.038±0.030	0.980±0.125
60	$6.77\times 10^{1}$	29.6±112.1	41.9±35.9	0.031±0.026	1.18±0.299
70	$1.31\times 10^{4}$	42.8±59.8	6.73±17.3	0.049±0.020	1.00±0.162

Temperature-dependent changes in absorption ($\mu_{a}$) and reduced scattering ($\mu_{s}^{\prime }$) coefficients of the porcine dermis in hemoglobin- and water-dominant spectral regions. Percent differences represent changes between pre- and post-treatment measurements (n=7 each, mean ± SD), with positive values indicating increases. Post-treatment absolute values are provided for comparison. Thermal damage is quantified using the Arrhenius damage integral (Ω).

The data presented in Table 1 demonstrates a clear relationship between the Arrhenius damage integral (Ω) and the temperature-dependent tissue changes described by Moxr and Henriques in their foundational work on thermal injury.^10^ The progression of Ω values from $2.52\times 10^{-8}$ at 22°C to $1.31\times 10^{4}$ at 70°C quantifies the exponential increase in thermal damage that occurs with rising temperatures. Our measurements of percent differences between pre- and post-treatment values reveal that minimal tissue alterations occur in the physiological temperature range (37°C to 43°C) where Ω remains below $3.38\times 10^{-3}$, mirroring their observation that tissue can tolerate moderate heating with limited permanent damage. A notable transition begins at 50°C, where Ω reaches 1.19 and optical properties begin to show negative percent differences (−5.59±35.3% for $\mu_{a}$), which may represent the initial stage of irreversible damage, though the large standard deviation suggests variability in this response. This is further amplified at 60°C, where Ω increases to $6.77\times 10^{1}$ and coincides with significant scattering coefficient changes (+40.9±36.7%), corresponding to their identified threshold where “irreversible cellular injury” rapidly accelerates. The subsequent changes in optical property trends at 70°C, where Ω reaches $1.31\times 10^{4}$, align with their concept of a “critical thermal threshold where tissue structural integrity becomes compromised.” This temperature-dependent behavior provides quantitative evidence for the thermal damage mechanisms described in their rate-process model of cutaneous injury.^10^

**Fig. 7:**
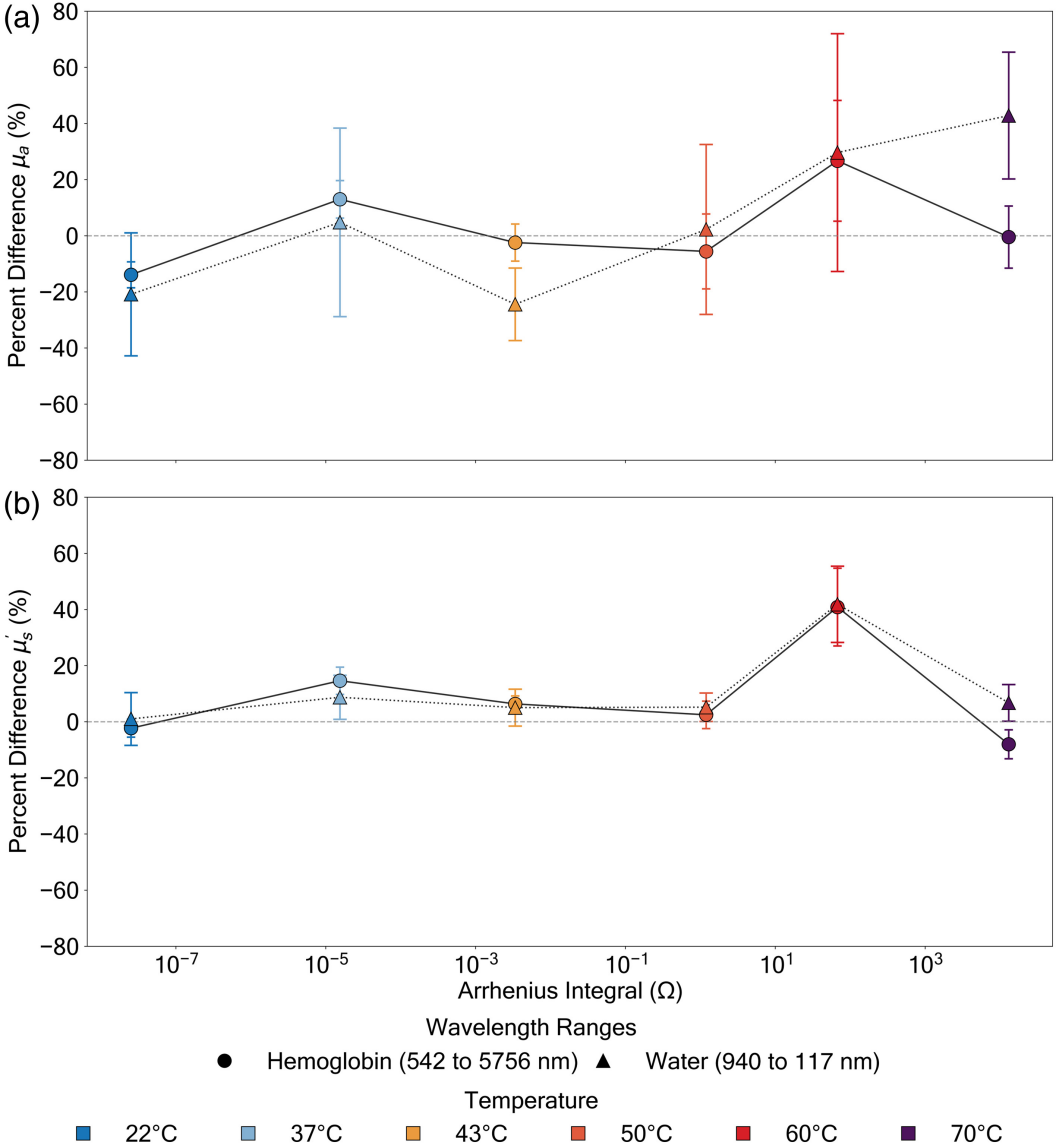
Percent difference in absorption coefficient ($\mu_{a}$) (a) and reduced scattering coefficient ($\mu_{s}^{\prime }$) (b) of the porcine dermis relative to baseline measurements as a function of the Arrhenius integral (Ω). Solid lines with circles represent the hemoglobin-dominant wavelength range (542 to 576 nm), whereas dotted lines with triangles show the water-dominant wavelength range (940 to 1170 nm). All values reported as mean ± SD (n=7) after outlier removal using z-score analysis (Sec. 2.4). The water-dominant region shows a decrease in initial percent difference in $\mu_{a}$ (−20.9±57.9%) compared with the hemoglobin region (−13.9±12.2%) at 22°C. Both wavelength regions exhibit distinct non-monotonic patterns in $\mu_{s}^{\prime }$, with maximum increases at 60°C ($\Omega =6.77\times 10^{1}$), reaching 40.9±36.7% and 41.9±35.9% for hemoglobin and water regions, respectively, followed by distinct changes at 70°C to −8.03±13.6% and +6.73±17.3%.

Examination of relative changes in optical properties provided additional insights into the relationship between thermal damage and tissue response (Fig. 7). Both absorption and scattering exhibited correlations with the Arrhenius integral. The absorption coefficient showed distinct phases of thermal response, with a change of −13.9±12.2% at room temperature in the hemoglobin region and a more pronounced decrease of −20.9±57.9% in the water-dominant region. These changes are moderated through the physiological range before reaching negative values at higher temperatures in the hemoglobin region.

The scattering coefficient demonstrated an even more striking non-monotonic pattern. At physiological temperatures, we observed increases of approximately +14.6±12.8% at 37°C and +6.35±7.58% at 43°C in the hemoglobin region. At denaturation temperatures, we first observed increases to approximately +40.9±36.7% at 60°C in the hemoglobin region and +41.9±35.9% in the water region, followed by a reversal to −8.03±13.6% at 70°C in the hemoglobin region while remaining positive (+6.73±17.3%) in the water region.

These well-defined, non-linear patterns of change—spanning Arrhenius integral values from $2.52\times 10^{-8}$ to $1.31\times 10^{4}$—suggest that tissue optical properties undergo predictable modifications during thermal treatment, with significant implications for both therapeutic applications and monitoring strategies.

### 3.3 Thermal Treatment Effects on Tissue Thickness

**Fig. 8:**
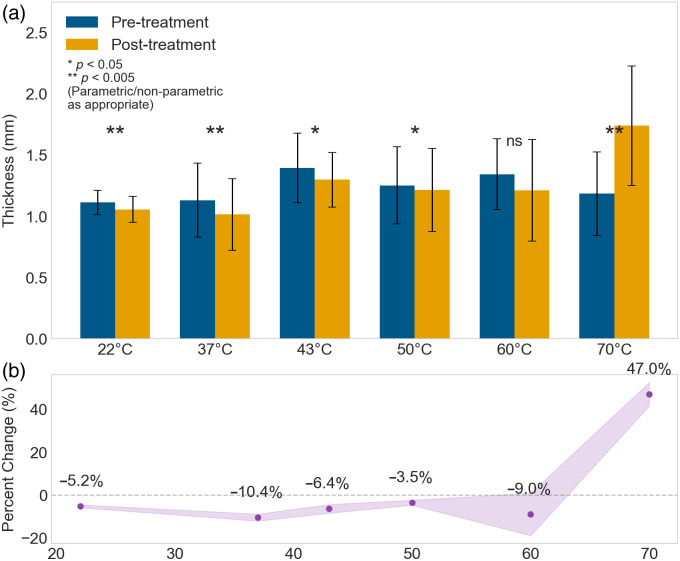
Temperature-dependent tissue thickness changes following thermal treatment. (a) Bar plot comparing pre-treatment (blue) and post-treatment (yellow) tissue thickness measurements (mean ± SD) across temperatures ranging from 22°C to 70°C. Statistical significance was determined using paired t-tests for normally distributed data and Wilcoxon signed-rank tests when normality assumptions were violated, with significance indicated by asterisks (*p<0.05 and **p<0.005; ns, not statistically significant). (b) Percent change in tissue thickness as a function of temperature, with shaded regions representing 95% confidence intervals.

Thermal treatment induced temperature-dependent changes in tissue thickness, with distinct patterns emerging across the temperature range (22°C to 70°C) (Fig. 8). These measurements were based on samples that remained after z-score analysis for outlier removal, resulting in n=7 paired measurements per temperature group. Statistical significance was determined using paired t-tests when normality assumptions were satisfied (Shapiro–Wilk test, p>0.05) and Wilcoxon signed-rank tests when normality was violated, ensuring appropriate statistical analysis despite the modest sample sizes.

At temperatures from 22°C to 60°C, tissues consistently showed compression, with thickness decreases ranging from −3.5% to −10.4%. The most pronounced compression occurred at 37°C (−10.4%, p<0.005), which was statistically significant. Other significant compressions were observed at 22°C (−5.2%, p<0.005), 43°C (−6.4%, p<0.05), and 50°C (−3.5%, p<0.05). At 60°C, despite showing substantial compression (−9.0%), the change was not statistically significant (p=0.58, Wilcoxon signed-rank test, as normality assumptions were violated for this condition).

A dramatic transition in tissue response occurred at 70°C, where tissues exhibited substantial expansion with a 47.0% increase in thickness (p<0.005). The lack of statistical significance at 60°C, despite showing substantial compression, reflects both increased variability in tissue response and the conservative nature of non-parametric testing as temperatures approach the critical transition point.

The analysis reveals increased variability in tissue response at 70°C, suggesting heterogeneous tissue reactions at this temperature. This dramatic shift from consistent compression across lower temperatures to marked expansion at 70°C indicates a fundamental change in tissue behavior, likely related to protein denaturation and structural changes in the extracellular matrix.^38^^–^^42^

From a methodological perspective, thinner samples were 45% more likely to be flagged as outliers during z-score analysis of optical properties. This relationship is consistent with expected signal-to-noise effects, where reduced sample thickness leads to fewer photon–tissue interactions, pushing measurements closer to the noise floor and reducing measurement reliability.

### 3.4 Discussion

The thermal-induced changes in tissue properties revealed complex mechanisms of modification that vary significantly with temperature, affecting both optical properties and physical dimensions. The non-linear behavior in absorption coefficients, transitioning from a 13.92±12.2% change at room temperature to a −5.59±35.3% decrease at 50°C, suggests progressive alterations in chromophore concentration and tissue structure, though the large standard deviations indicate substantial sample-to-sample variability. These changes could be attributed to protein denaturation and subsequent modifications in the molecular cross-sections of primary absorbers such as hemoglobin and water, alongside initial structural reorganization.

The non-linear response of the reduced scattering coefficient, particularly the dramatic changes at 60°C and 70°C, suggests a complex sequence of structural modifications. The remarkable peak in scattering at 60°C (+40.87±36.65% in the hemoglobin region) likely represents the formation of denatured protein aggregates, increasing the number of scattering centers. The subsequent change at 70°C (−8.03±13.64% in the hemoglobin region and +6.73±17.28% in the water region) may indicate structural collapse or homogenization of these aggregates, with wavelength-dependent effects. The Arrhenius integral analysis supports this interpretation, which shows an exponential increase with temperature from $1.55\times 10^{-5}$ at 37°C to $1.31\times 10^{4}$ at 70°C.

The wavelength-dependent responses in optical properties provide comprehensive insights into the specific mechanisms of thermal modification. The enhanced changes in the near-infrared region (900 to 1000 nm) for absorption suggest particular sensitivity of water-based chromophores to thermal stress. The pronounced scattering changes in the visible spectrum (400 to 600 nm) indicate that thermal modification primarily affects structures on the scale of visible wavelengths, consistent with collagen fiber networks and cellular components.

The relationship between thermal damage accumulation and tissue property changes demonstrates intriguing complexity. The temperature-dependent changes in absorption coefficient suggest that thermal damage systematically affects tissue chromophores. The non-monotonic response pattern for scattering coefficient changes indicates that structural modifications follow well-defined but complex kinetics involving multiple competing processes.

Our results in the hemoglobin region reveal distinct non-linear trends that differ from prior reports of more gradual thermal responses. For example, Laufer et al.^18^ reported a linear increase in scattering with temperature, whereas we observed a pronounced peak at 60°C ($\Delta \mu_{s}^{\prime }=+40.87\pm 36.65\%$), followed by a decrease at 70°C (−8.03±13.64%), suggesting threshold-based structural transitions such as protein aggregation and collapse. Similarly, although Iorizzo et al.^19^ and Barton et al.^17^ observed increasing absorption with temperature, our data show variable trends including a reduction in absorption at elevated temperatures (e.g., $\Delta \mu_{a}=-5.59\pm 35.3\%$ at 50°C). These differences point to complex, non-monotonic mechanisms not fully captured by earlier models, emphasizing the need for expanded thermal response frameworks in light–tissue interaction studies.

These findings have significant implications for therapeutic applications. The correlation between thermal damage and optical property changes could serve as potential metrics for real-time monitoring of treatment progression. The wavelength-dependent variations and temperature-specific structural changes suggest opportunities for protocol optimization, potentially allowing for selective targeting of specific tissue modifications while minimizing unwanted effects. Furthermore, the identification of distinct temperature thresholds, particularly around 60 °C where tissue properties begin to change dramatically, provides valuable guidance for treatment protocol development.

The observed transitions in tissue properties align with known thermally-induced changes in structure. The moderate changes observed in the physiological temperature range (37°C to 43°C), characterized by increases in scattering ($\Delta \mu_{s}^{\prime }=+14.61\pm 12.82\%$ at 37°C and +6.35±7.58% at 43°C in the hemoglobin region) and varying absorption responses, correspond to reversible protein modifications.^38^^–^^42^ The pronounced changes at higher temperatures coincide with irreversible denaturation processes. The complex behavior at 60°C and 70°C, characterized by dramatic shifts in optical properties, suggests multiple competing mechanisms, including protein aggregation, structural collapse, and potential tissue carbonization.^38^^–^^42^ These comprehensive changes in tissue properties provide a more complete understanding of thermal modification mechanisms and their implications for therapeutic applications.

## 4 Conclusion

This study has characterized the complex temperature-dependent changes in optical properties of porcine dermis across a thermal exposure range from 22°C to 70°C. Our findings demonstrate that thermal treatment induces significant, non-linear alterations in both absorption coefficient ($\mu_{a}$) and reduced scattering coefficient ($\mu_{s}^{\prime }$) that vary with wavelength and follow distinct patterns depending on the degree of thermal damage.

The observed changes in optical properties correlate with the Arrhenius damage integral (Ω), providing quantitative evidence for the thermal damage mechanisms described in classical rate-process models of cutaneous injury. In the physiological temperature range (37°C to 43°C), where Ω remains below $3.38\times 10^{-3}$, tissue demonstrated relatively stable absorption characteristics ($\Delta \mu_{a}=+4.64\pm 3.46\%$ at 37°C) with moderate increases in scattering ($\Delta \mu_{s}^{\prime }=+17.28\pm 8.33\%$). As temperatures increased to the denaturation threshold at 60°C ($\Omega =6.77\times 10^{1}$), we observed a marked transition characterized by decreased absorption in the hemoglobin region ($\Delta \mu_{a}=-15.87\pm 6.12\%$) and dramatically increased scattering ($\Delta \mu_{s}^{\prime }=+43.58\pm 10.11\%$). At 70°C ($\Omega =1.31\times 10^{4}$), the reversal in scattering trends suggests further structural breakdown, with wavelength-dependent effects indicating complex mechanisms.

The tissue thickness measurements provided complementary insights, revealing a transition from mild compression at lower temperatures (−10.4% to −3.5% at 22°C to 60°C) to significant expansion at higher temperatures (+47.0% at 70°C, p<0.005). This pattern aligns with the non-monotonic behavior observed in optical properties.

### 4.1 Assessment of Methods

The experimental approach using controlled thermal exposures (2.5 min) across six temperature points (22°C to 70°C) enabled systematic characterization of optical property changes. The broad spectral measurement range (450 to 1050 nm) allowed for examination of both hemoglobin-dominant and water-dominant regions, though higher variability was observed in the water region. Integration of the Arrhenius damage model (Ω values spanning $2.52\times 10^{-8}$ to $1.31\times 10^{4}$) effectively quantified thermal damage accumulation and its relationship to optical property changes. Future methodological considerations should include investigation of exposure-time dependence and assessment of reversibility in transitional temperature zones.

The use of 0.9% normal saline solution for sample preparation warrants consideration regarding its potential impact on tissue blood content and hemoglobin absorption characteristics. Although the 15-min saline immersion was designed to standardize hydration levels across samples, this process may have resulted in partial removal or dilution of residual blood components within the dermis tissue. Any reduction in blood content due to saline washing could potentially affect the baseline optical properties and the magnitude of thermal-induced changes observed in this spectral region. However, the consistent saline treatment protocol applied across all temperature groups ensures that any blood-related effects would be uniformly distributed, allowing for valid comparative analysis of thermal treatment effects. Future studies may benefit from investigating the specific impact of saline immersion duration on residual blood content and its influence on hemoglobin-related absorption features, particularly when studying tissues with higher vascular density or when hemoglobin content is a primary parameter of interest.

The tissue storage protocol employed in this study involved initial freezing at −20°C followed by refrigeration at 4°C for 1 to 5 h prior to optical measurements. This storage sequence warrants consideration in light of recent findings by Hoffman et al.^30^ who systematically investigated the effects of cold storage on porcine dermis optical properties using a similar double-integrating sphere methodology. Their study demonstrated that freezing at −18°C for 1 week resulted in average changes of $-0.003\;{\mathrm{mm}}^{-1}$) in absorption coefficient and $-0.001\;{\mathrm{mm}}^{-1}$) in reduced scattering coefficient for the dermis, with the most notable differences occurring in the hemoglobin absorption region (400 to 600 nm). In addition, refrigeration at 4°C for 24 h produced changes of $-0.005\;{\mathrm{mm}}^{-1}$) in absorption coefficient and $-0.05\;{\mathrm{mm}}^{-1}$) in reduced scattering coefficient. Although our refrigeration period was substantially shorter (1 to 5 h versus 24 h), the baseline optical properties measured at 22°C in our study likely reflect some degree of storage-induced modification compared with truly fresh tissue. However, as all samples in our investigation underwent identical storage protocols, any storage-related effects should be uniformly distributed across all temperature groups, preserving the validity of the comparative thermal damage analysis.

Future work would benefit from incorporating histological analysis to provide complementary structural information about the thermal damage mechanisms underlying the observed optical property changes. Such microscopic examination could offer valuable insights into the relationship between tissue morphology and spectral alterations, potentially enhancing our understanding of the physical processes driving these optical responses.

These findings have important implications for optical imaging and therapeutic applications that rely on specific spectral windows. The identified temperature thresholds and corresponding changes in optical properties can inform protocol development for thermal therapies, potentially enabling real-time monitoring of treatment progression through optical measurements. Future work should explore the reversibility of these changes, particularly in the transitional temperature zones, and investigate their impact on therapeutic efficacy in clinical settings.

In conclusion, our comprehensive characterization of temperature-dependent optical property changes provides a foundation for optimizing thermal treatments and developing more accurate predictive models of light–tissue interactions during therapeutic interventions. The non-linear relationships among temperature, Arrhenius damage, and optical properties underscore the complexities of thermal damage mechanisms in biological tissues.

## Supplementary Material

10.1117/1.JBO.30.10.105003.s01

## Data Availability

The data used in this study are freely available upon reasonable request to the authors.
