# Supplementary material for: Thermal damage induced changes in optical properties of the porcine dermis
Source: J Biomed Opt. 2025 Oct 11;30(10):105003. doi: 10.1117/1.JBO.30.10.105003 (PMC12515067; doi:10.1117/1.JBO.30.10.105003)
Supplement: Supplementary file 1 [file JBO_030_105003_SD001.pdf]

Supplementary Materials:

Thermal damage induced changes in optical properties of porcine dermis from 400 to 1100 nm

Anjelyka Fasci[a,b], **Maria A. Troyanova-Wood**[a], **Andrea L. Smith**[a], **Matthew E. Macasadia**[a], **Amanda J. Tijerina**[c], **R. Lyle Hood**[b], **Michael P. DeLisi**[a], **Joel N. Bixler**[d,e*]
[a]SAIC, JBSA Fort Sam Houston, Texas 78234
[b]University of Texas at San Antonio, Department of Mechanical Engineering, San Antonio, Texas 78249
[c]Conceptual Mindworks, Inc., San Antonio, Texas 78234
[d]Texas A&M University, Department of Biomedical Engineering, College Station, Texas 77843
[e]Air Force Research Laboratory, JBSA Fort Sam Houston, Texas 78234

1 BioPixS004 Optical Tissue Phantom

To establish an appropriate calibration technique and confirm system stability prior to tissue optical property measurements, a BioPix optical phantom (BioPixS0064, BioPixS Ltd., Cork, Ireland) was measured multiple times over several days. The BioPix phantom serves as a stable reference standard with known optical properties, allowing for validation of measurement accuracy and precision. Data for 20 different measurements across three wavelengths is shown in Fig (1) and demonstrates stable performance, validating the system's reliability for subsequent tissue measurements. This calibration approach ensures that any variations observed in tissue optical properties can be attributed to actual biological differences rather than instrumental variability, providing confidence in the measurement system's precision and accuracy throughout the experimental protocol.

For the absorption coefficient (μ_a), measurements at 690 nm showed a mean of 0.0826 ± 0.0049 mm^{-1} with a coefficient of variation (CV) of 12.75%. The μ_a values decreased with longer wavelengths, with means of 0.0705 ± 0.0053 mm^{-1} at 785 nm and 0.0684 ± 0.0054 mm^{-1} at 830 nm, while showing slightly higher variability (CVs of 16.02% and 16.89% respectively). The reduced scattering coefficient (μ_s') demonstrated superior stability across all wavelengths, with CVs consis-

tently below 8%. The μ_s' values showed the expected decrease with increasing wavelength, from 1.1626 ± 0.0309 mm^{-1} at 690 nm to 1.0002 ± 0.0336 mm^{-1} at 785 nm and 0.9453 ± 0.0322 mm^{-1} at 830 nm. The substantially lower variability in the reduced scattering coefficient measurements (5.71-7.29%) compared to the absorption coefficient measurements (12.75-16.89%) indicates that the scattering properties of the phantom remained more stable, demonstrating reliable system performance for both absorption and scattering measurements with particularly robust scattering characterization.

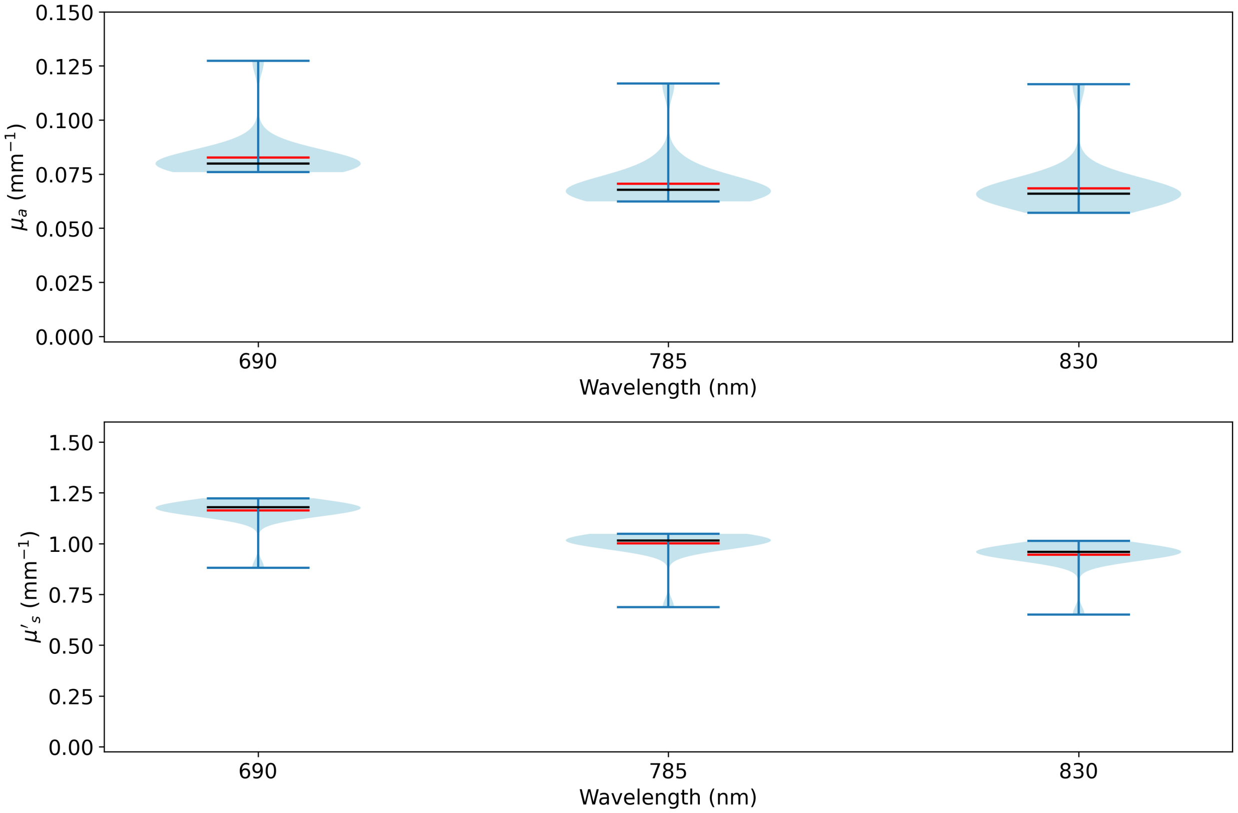

Fig 1 Optical properties of the BioPixS0064 phantom measured across three wavelengths ($n = 20$). The figure displays violin plots showing the distribution of absorption coefficient (μ_a, top) and reduced scattering coefficient (μ'_s, bottom) at 690, 785, and 830 nm wavelengths. Each violin plot combines the probability density (shaded area) with box plot elements, where black horizontal lines indicate the median and red lines show the mean values. The absorption coefficient demonstrates wavelength-dependent behavior, decreasing from 0.0826 ± 0.0049 mm^{-1} at 690 nm to 0.0705 ± 0.0053 mm^{-1} at 785 nm and 0.0684 ± 0.0054 mm^{-1} at 830 nm (mean ± 95% CI). Similarly, the reduced scattering coefficient shows a decreasing trend with increasing wavelength: 1.1626 ± 0.0309 mm^{-1} (690 nm), 1.0002 ± 0.0336 mm^{-1} (785 nm), and 0.9453 ± 0.0322 mm^{-1} (830 nm). The reduced scattering coefficient exhibits lower variability (coefficient of variation: 5.71-7.29%) compared to the absorption coefficient (12.75-16.89%), indicating more consistent measurements across the sample population.

Table S1: Pre- and post-treatment tissue thickness measurements (mm) across temperature conditions. Data shows estimated tissue thickness before (Pre) and after (Post) thermal treatment at six different temperatures. Measurements are corrected for glass slide thickness by removing 2 glass slides (2×1.0533 mm = 2.1066 mm total) from original measurements. Samples removed by z-score analysis are marked in red with †. Statistical significance: * $p < 0.05$, ** $p < 0.005$, ns = not significant.

Sample	**22°C**** (n=7)		**37°C**** (n=7)		**43°C*** (n=7)		**50°C*** (n=7)		**60°C**^{ns} (n=7)		**70°C**** (n=7)	
	Pre	Post	Pre	Post	Pre	Post	Pre	Post	Pre	Post	Pre	Post
1	1.1049	1.0109	1.2184	1.0434	1.5884†	1.5644†	0.9884†	0.8784†	1.0154	1.1184	1.0134†	1.4884†
2	1.1814	1.1359	1.0534	0.9614	1.1744	1.1584	1.4234	1.3634	1.2194†	1.3684†	0.9654	1.3680
3	1.1834	1.1334	1.1184†	0.9934†	1.2584	1.1784	1.0534†	1.0284†	1.2114†	1.2534†	1.4414†	2.2708†
4	1.1659	1.1284	0.9584†	0.9684†	1.8534	1.6034	1.2634†	1.2384†	1.3254†	1.4054†	1.6534	2.3072
5	1.1624	1.1214	0.8234	0.7784	1.2084†	1.0484†	1.0514	0.9534	0.6824†	0.6624†	1.2414	1.7708
6	1.1704†	0.9434†	0.8634†	0.7134†	1.0134	0.9684	1.2334	1.2134	1.1654†	1.2334†	0.7284	0.9898
7	0.9069	0.8504	1.3034	1.1784	1.3284	1.2384	1.6934	1.7184	1.6084	1.6934	1.5434	2.2876
8	0.9099†	0.8614†	1.0984†	1.0514†	0.7584†	0.7524†	1.5484	1.5254	1.5984†	1.7424†	1.2644	1.9600
9	1.0819	1.0109	0.9634	0.8884	1.5754	1.4754	0.8684	0.8414	1.6434	1.7534	0.8984	1.4926
10	1.1384†	1.2984†	1.6884	1.5684	1.5584	1.4684	0.9434	0.8854	1.0584	1.1584	1.5534†	2.3556†
11	—	—	0.8644	0.6914	0.6084†	0.5424†	—	—	1.6154	1.2644	—	—
12	—	—	—	—	—	—	—	—	0.6844†	1.2844†	—	—
13	—	—	—	—	—	—	—	—	1.0684	0.9224	—	—
14	—	—	—	—	—	—	—	—	1.3954	0.5724	—	—
15	—	—	—	—	—	—	—	—	1.4554†	1.3314†	—	—

† Samples removed by outlier analysis. Statistical significance based on kept samples only.

2 Mean Absorption Coefficient across Spectra

The absorption coefficient (μ_a) values presented in Table S2 represent the mean optical properties measured across the wavelength range of 450-1170 nm at 10 nm intervals. Measurements were obtained at six different temperature conditions (22°C, 37°C, 43°C, 50°C, 60°C, and 70°C) under both pre-treatment and post-treatment experimental conditions. Statistical significance between pre- and post-treatment values was assessed using paired t-tests, with significance levels indicated by * ($p < 0.05$) and ** ($p < 0.01$). The data reveals temperature-dependent changes in tissue absorption properties, with notable significant differences observed particularly at higher temperatures and in the near-infrared wavelength regions.

Table S2: Average values of absorption coefficient (μ_a)

λ, nm	22°C			37°C			43°C			50°C			60°C			70°C		
	Pre	Post	sig.	Pre	Post	sig.	Pre	Post	sig.	Pre	Post	sig.	Pre	Post	sig.	Pre	Post	sig.
450	0.101	0.082		0.083	0.079		0.079	0.074		0.088	0.079	**	0.074	0.093		0.063	0.073	
460	0.091	0.077		0.074	0.071		0.074	0.066		0.083	0.078	**	0.067	0.084		0.058	0.065	
470	0.087	0.073		0.071	0.073		0.067	0.065		0.081	0.073	**	0.062	0.076		0.053	0.060	
480	0.082	0.066		0.065	0.067		0.064	0.061		0.076	0.073	*	0.060	0.074		0.053	0.061	
490	0.079	0.067		0.064	0.065		0.061	0.060		0.076	0.073		0.056	0.070		0.048	0.056	
500	0.076	0.064		0.060	0.065		0.059	0.057		0.071	0.070		0.054	0.069		0.047	0.053	
510	0.074	0.064		0.059	0.063		0.058	0.056		0.070	0.068		0.053	0.067		0.047	0.050	
520	0.073	0.064		0.058	0.063		0.057	0.055		0.073	0.068	*	0.051	0.067		0.048	0.051	
530	0.076	0.066		0.060	0.067		0.059	0.060		0.075	0.070		0.052	0.068		0.050	0.052	
540	0.080	0.067		0.062	0.069		0.062	0.059		0.077	0.073		0.053	0.069		0.052	0.052	
550	0.078	0.067		0.058	0.065		0.060	0.057		0.075	0.069	*	0.050	0.065		0.050	0.050	
560	0.073	0.064		0.058	0.062		0.057	0.059		0.072	0.069		0.049	0.062		0.047	0.049	
570	0.077	0.068		0.058	0.066		0.061	0.059		0.074	0.071		0.050	0.061		0.050	0.049	
580	0.075	0.067		0.059	0.067		0.059	0.059		0.076	0.074		0.049	0.063		0.051	0.049	
590	0.071	0.061		0.052	0.062		0.055	0.054		0.069	0.067		0.044	0.057		0.047	0.046	
600	0.068	0.059		0.050	0.060		0.055	0.054		0.068	0.065		0.042	0.055		0.046	0.044	
610	0.067	0.059		0.049	0.061		0.054	0.054		0.067	0.065		0.041	0.054		0.045	0.044	
620	0.067	0.058		0.048	0.060		0.053	0.053		0.066	0.064		0.042	0.053		0.046	0.043	
630	0.065	0.060		0.048	0.059		0.053	0.055		0.065	0.064		0.040	0.051		0.046	0.043	
640	0.066	0.060		0.048	0.058		0.052	0.054		0.066	0.064		0.038	0.050		0.046	0.044	
650	0.065	0.058		0.044	0.057		0.054	0.054		0.064	0.064		0.038	0.049		0.046	0.042	
660	0.063	0.058		0.043	0.057		0.053	0.054		0.064	0.063		0.037	0.048		0.046	0.043	
670	0.064	0.056		0.042	0.057		0.052	0.054		0.065	0.062		0.035	0.046		0.045	0.043	
680	0.062	0.056		0.041	0.056		0.053	0.054		0.062	0.061		0.035	0.045		0.044	0.042	
690	0.061	0.056		0.040	0.053		0.052	0.054		0.061	0.062		0.034	0.045		0.044	0.042	
700	0.061	0.054		0.038	0.051		0.051	0.054		0.061	0.060		0.033	0.042		0.041	0.042	

Table S2: Average values of absorption coefficient (μ_a)

	22°C			37°C			43°C			50°C			60°C			70°C		
λ, nm	Pre	Post	sig.	Pre	Post	sig.	Pre	Post	sig.	Pre	Post	sig.	Pre	Post	sig.	Pre	Post	sig.
710	0.058	0.054		0.037	0.051		0.052	0.054		0.060	0.058		0.032	0.040		0.041	0.041	
720	0.057	0.054		0.035	0.049		0.052	0.052		0.059	0.057		0.032	0.040		0.040	0.040	
730	0.057	0.051		0.032	0.047		0.050	0.053		0.057	0.057		0.031	0.039		0.039	0.041	
740	0.056	0.050		0.030	0.047		0.050	0.051		0.057	0.055		0.029	0.038		0.039	0.039	
750	0.056	0.051		0.029	0.044		0.050	0.052		0.056	0.054		0.028	0.038		0.038	0.039	
760	0.054	0.048		0.027	0.042		0.050	0.050		0.055	0.055		0.028	0.037		0.038	0.039	
770	0.052	0.048		0.027	0.040		0.049	0.051		0.052	0.053		0.026	0.036		0.035	0.041	
780	0.051	0.045		0.026	0.040		0.048	0.050		0.052	0.050		0.025	0.033		0.035	0.038	
790	0.050	0.045		0.026	0.038		0.047	0.050		0.050	0.050		0.025	0.033		0.033	0.037	
800	0.051	0.044		0.025	0.037		0.048	0.049		0.050	0.050		0.025	0.033		0.033	0.037	
810	0.050	0.044		0.025	0.036		0.046	0.047		0.047	0.048		0.023	0.033		0.032	0.037	
820	0.048	0.043		0.025	0.035		0.047	0.048		0.048	0.047		0.023	0.032		0.032	0.037	
830	0.049	0.041		0.024	0.035		0.045	0.047		0.047	0.047		0.022	0.031		0.032	0.038	
840	0.047	0.043		0.024	0.034		0.047	0.047		0.048	0.047		0.023	0.032	$*$	0.032	0.037	
850	0.046	0.042		0.023	0.034		0.047	0.048		0.047	0.047		0.022	0.031	$*$	0.030	0.037	
860	0.045	0.041		0.022	0.035		0.046	0.046		0.044	0.046		0.021	0.031	$*$	0.030	0.037	
870	0.046	0.042		0.022	0.034		0.047	0.048		0.045	0.045		0.020	0.030	$*$	0.030	0.037	
880	0.045	0.041		0.022	0.033		0.046	0.048		0.045	0.045		0.020	0.029	$*$	0.030	0.038	
890	0.044	0.038		0.022	0.033		0.046	0.048		0.046	0.045		0.020	0.029	$*$	0.030	0.037	
900	0.046	0.039		0.022	0.032		0.047	0.048		0.044	0.045		0.020	0.030	$*$	0.029	0.038	
910	0.046	0.039		0.022	0.031		0.048	0.049		0.044	0.046		0.020	0.031	$*$	0.029	0.037	
920	0.046	0.042		0.022	0.031		0.048	0.048		0.043	0.045		0.020	0.029	$*$	0.029	0.040	
930	0.047	0.042		0.023	0.031		0.049	0.049		0.044	0.046		0.022	0.031	$*$	0.030	0.040	
940	0.049	0.045		0.025	0.035		0.051	0.053		0.048	0.048		0.024	0.034	$*$	0.035	0.045	
950	0.052	0.048		0.028	0.040		0.059	0.061		0.052	0.053		0.026	0.038	$*$	0.039	0.052	
960	0.064	0.053		0.032	0.047	$*$	0.066	0.068		0.059	0.059		0.032	0.045	$*$	0.050	0.063	

Table S2: Average values of absorption coefficient (μ_a)

	22°C			37°C			43°C			50°C			60°C			70°C		
λ, nm	Pre	Post	sig.	Pre	Post	sig.	Pre	Post	sig.	Pre	Post	sig.	Pre	Post	sig.	Pre	Post	sig.
970	0.065	0.055		0.033	0.049	*	0.070	0.070		0.062	0.062		0.033	0.046	*	0.053	0.066	
980	0.064	0.055		0.034	0.047		0.070	0.071		0.063	0.061		0.034	0.045	*	0.051	0.068	
990	0.062	0.053		0.032	0.046	*	0.069	0.071		0.059	0.059		0.033	0.043		0.048	0.065	
1000	0.059	0.050		0.033	0.045		0.066	0.064		0.054	0.056		0.029	0.039		0.043	0.060	
1010	0.056	0.048		0.029	0.038		0.062	0.063		0.052	0.050		0.028	0.040	*	0.040	0.057	
1020	0.048	0.041		0.027	0.037		0.057	0.056		0.050	0.049		0.023	0.035	*	0.036	0.053	
1030	0.047	0.037		0.022	0.035		0.057	0.055		0.041	0.044		0.022	0.032		0.034	0.048	
1040	0.042	0.032		0.020	0.030		0.053	0.049		0.039	0.040		0.020	0.028		0.031	0.043	
1050	0.038	0.027		0.018	0.029		0.050	0.048		0.035	0.037		0.018	0.025		0.029	0.043	
1060	0.036	0.023		0.016	0.029	*	0.049	0.045		0.031	0.032		0.018	0.023		0.027	0.040	
1070	0.036	0.026		0.017	0.025		0.047	0.040		0.030	0.031		0.015	0.020		0.025	0.038	
1080	0.031	0.020		0.023	0.033		0.052	0.040		0.026	0.030		0.019	0.020		0.023	0.038	*
1090	0.031	0.014		0.017	0.029		0.049	0.047		0.027	0.030		0.018	0.023		0.027	0.036	
1100	0.025	0.013		0.022	0.026		0.043	0.051		0.031	0.024		0.014	0.023		0.017	0.037	**
1110	0.018	0.008		0.033	0.028		0.067	0.038	*	0.020	0.020		0.014	0.022		0.018	0.026	
1120	0.015	0.009		0.022	0.032		0.040	0.040		0.026	0.019		0.024	0.024		0.028	0.033	
1130	0.027	0.012		0.042	0.044		0.082	0.038	**	0.027	0.025		0.021	0.022		0.027	0.038	
1140	0.013	0.011		0.029	0.039		0.154	0.071	**	0.014	0.022		0.022	0.032		0.037	0.035	
1150	0.007	0.009		0.063	0.097	*	0.082	0.075		0.028	0.019		0.026	0.020		0.029	0.063	**
1160	0.025	0.017		0.115	0.042	**	0.275	0.061	**	0.014	0.013		0.020	0.034		0.017	0.061	**
1170	0.010	0.017		0.250	0.075	**	0.360	0.102		0.020	0.021		0.009	0.006		0.022	0.062	**

3 Mean Reduced Scattering Coefficient across Spectra

Table S3 presents the reduced scattering coefficient ($\mu_s{}'$) measurements obtained under identical experimental conditions as the absorption coefficient data. The reduced scattering coefficient characterizes the light scattering properties of the tissue, accounting for the anisotropy of scattering events. Measurements span the same wavelength range (450-1170 nm) and temperature conditions (22°C - 70°C), comparing pre-treatment and post-treatment values. Statistical analysis using paired t-tests reveals significant temperature-induced changes in scattering properties, with the most pronounced effects observed at physiological and hyperthermic temperatures. The scattering coefficient generally decreases with increasing wavelength, following the expected wavelength dependence of Mie scattering in biological tissues.

Table S3: Average values of reduced scattering coefficient (μ_s')

	22°C			37°C			43°C			50°C			60°C			70°C		
λ, nm	Pre	Post	sig.	Pre	Post	sig.	Pre	Post	sig.	Pre	Post	sig.	Pre	Post	sig.	Pre	Post	sig.
450	6.619	6.522		6.372	6.987		6.432	6.688		6.895	7.106		5.227	6.786	*	7.564	5.309	**
460	6.402	6.286		5.705	6.555	**	6.034	6.464		6.508	6.720		4.957	6.453	*	6.944	5.224	**
470	6.030	5.873		5.425	6.258	**	5.545	6.095	*	6.134	6.340		4.661	6.154	*	6.665	5.061	**
480	5.596	5.561		5.079	5.858	**	5.290	5.653		5.795	5.900		4.381	5.938	*	6.191	4.834	**
490	5.314	5.247		4.758	5.472	**	4.983	5.403	*	5.429	5.564		4.177	5.604	*	5.835	4.668	**
500	5.014	4.985		4.469	5.115	**	4.733	5.105		5.012	5.242		3.963	5.370	*	5.505	4.560	*
510	4.767	4.700		4.183	4.803	**	4.440	4.753		4.693	4.880		3.723	5.150	*	5.095	4.357	*
520	4.551	4.449		3.904	4.483	**	4.215	4.481		4.439	4.569		3.531	4.908	*	4.771	4.139	*
530	4.286	4.184		3.668	4.226	**	3.974	4.240		4.192	4.321		3.343	4.636	*	4.536	3.933	*
540	4.131	3.983		3.478	4.031	**	3.796	4.055		4.009	4.088		3.181	4.464	*	4.261	3.794	
550	3.864	3.773		3.281	3.802	**	3.599	3.850		3.802	3.882		3.046	4.245	*	4.028	3.651	
560	3.709	3.610		3.123	3.593	**	3.418	3.664		3.575	3.648		2.905	4.053	*	3.842	3.509	
570	3.525	3.423		2.980	3.397	**	3.244	3.496	*	3.377	3.483		2.749	3.890	*	3.644	3.374	
580	3.368	3.279		2.853	3.261	**	3.135	3.351	*	3.223	3.311		2.643	3.731	*	3.410	3.234	
590	3.232	3.130		2.718	3.112	**	2.999	3.165		3.079	3.180		2.519	3.607	*	3.273	3.099	
600	3.091	3.012		2.591	2.960	**	2.866	3.047		2.961	3.006		2.421	3.442	*	3.113	2.988	
610	2.959	2.910		2.472	2.835	**	2.747	2.945	*	2.795	2.867		2.328	3.312	*	2.984	2.889	
620	2.860	2.775		2.373	2.722	*	2.652	2.812		2.701	2.763		2.224	3.200	*	2.860	2.785	
630	2.732	2.664		2.279	2.597	**	2.541	2.716	*	2.587	2.639		2.135	3.073	*	2.728	2.733	
640	2.643	2.586		2.180	2.493	**	2.468	2.601		2.482	2.548		2.062	2.975	*	2.628	2.604	
650	2.536	2.489		2.093	2.390	*	2.390	2.519		2.385	2.445		1.994	2.900	*	2.510	2.518	
660	2.453	2.396		2.026	2.311	*	2.305	2.435		2.299	2.359		1.929	2.778	*	2.417	2.472	
670	2.359	2.303		1.948	2.212	*	2.206	2.347		2.205	2.259		1.841	2.693	*	2.316	2.364	
680	2.270	2.218		1.866	2.130	**	2.137	2.268		2.132	2.194		1.781	2.606	*	2.233	2.291	
690	2.191	2.154		1.802	2.067	**	2.091	2.186		2.062	2.108		1.726	2.509	*	2.132	2.208	

Table S3: Average values of reduced scattering coefficient (μ_s')

	22°C			37°C			43°C			50°C			60°C			70°C		
λ, nm	Pre	Post	sig.	Pre	Post	sig.	Pre	Post	sig.	Pre	Post	sig.	Pre	Post	sig.	Pre	Post	sig.
700	2.109	2.062		1.737	1.973	$*$	2.003	2.114		1.967	2.030		1.667	2.429	$*$	2.062	2.129	
710	2.042	2.000		1.663	1.913	$**$	1.948	2.037		1.916	1.954		1.603	2.343	$*$	1.982	2.077	
720	1.963	1.931		1.617	1.857	$**$	1.877	1.963		1.844	1.888		1.558	2.262	$*$	1.909	2.003	
730	1.902	1.867		1.566	1.788	$**$	1.816	1.913		1.777	1.821		1.505	2.206	$*$	1.846	1.943	
740	1.852	1.821		1.514	1.723	$*$	1.760	1.832		1.718	1.759		1.456	2.131	$*$	1.776	1.869	
750	1.785	1.763		1.471	1.674	$*$	1.720	1.794		1.665	1.709		1.414	2.067	$*$	1.726	1.820	
760	1.739	1.708		1.426	1.627	$*$	1.667	1.740		1.618	1.657		1.372	2.015	$*$	1.673	1.763	
770	1.703	1.670		1.379	1.576	$**$	1.633	1.694		1.565	1.617		1.340	1.950	$*$	1.619	1.725	
780	1.647	1.627		1.342	1.533	$**$	1.590	1.658		1.531	1.568		1.306	1.902	$*$	1.576	1.675	
790	1.614	1.583		1.310	1.498	$**$	1.546	1.617		1.485	1.534		1.269	1.859	$*$	1.542	1.631	
800	1.568	1.551		1.282	1.462	$**$	1.523	1.577		1.448	1.490		1.236	1.814	$*$	1.498	1.591	
810	1.528	1.518		1.250	1.432	$**$	1.470	1.534		1.406	1.451		1.209	1.770	$*$	1.448	1.555	
820	1.497	1.466		1.223	1.392	$**$	1.441	1.500		1.369	1.407		1.183	1.728	$*$	1.414	1.524	
830	1.462	1.439		1.189	1.361	$**$	1.404	1.468		1.338	1.374		1.153	1.700	$*$	1.383	1.473	
840	1.425	1.410		1.175	1.331	$*$	1.385	1.430		1.302	1.355		1.124	1.650	$*$	1.348	1.457	
850	1.392	1.389		1.143	1.305	$*$	1.351	1.396		1.270	1.322		1.112	1.618	$*$	1.310	1.416	
860	1.372	1.355		1.122	1.279	$*$	1.325	1.369		1.252	1.292		1.090	1.574	$*$	1.280	1.381	
870	1.342	1.329		1.095	1.245	$*$	1.298	1.344		1.219	1.257		1.069	1.550	$*$	1.261	1.348	
880	1.321	1.300		1.070	1.219	$*$	1.266	1.324		1.195	1.241		1.047	1.525	$*$	1.229	1.330	
890	1.291	1.279		1.049	1.191	$*$	1.242	1.293		1.168	1.212		1.021	1.492	$*$	1.198	1.297	
900	1.270	1.253		1.014	1.167	$*$	1.227	1.260		1.146	1.180		1.001	1.454	$*$	1.170	1.267	
910	1.248	1.235		1.009	1.151	$**$	1.200	1.246		1.122	1.166		0.987	1.435	$*$	1.156	1.240	
920	1.223	1.222		0.986	1.133	$*$	1.176	1.214		1.107	1.134		0.967	1.404	$*$	1.133	1.216	
930	1.202	1.195		0.974	1.115	$*$	1.155	1.198		1.080	1.117		0.954	1.371	$*$	1.104	1.193	
940	1.177	1.181		0.951	1.086	$**$	1.136	1.180		1.061	1.102		0.933	1.346	$*$	1.094	1.170	

Table S3: Average values of reduced scattering coefficient (μ_s')

	22°C			37°C			43°C			50°C			60°C			70°C		
λ, nm	Pre	Post	sig.	Pre	Post	sig.	Pre	Post	sig.	Pre	Post	sig.	Pre	Post	sig.	Pre	Post	sig.
950	1.159	1.154		0.939	1.065	**	1.125	1.160		1.039	1.085		0.927	1.325	*	1.064	1.147	
960	1.145	1.142		0.928	1.055	**	1.100	1.137		1.030	1.064		0.911	1.316	*	1.042	1.126	
970	1.130	1.132		0.915	1.038	**	1.086	1.114		1.012	1.049		0.898	1.286	*	1.019	1.106	
980	1.114	1.114		0.907	1.016	*	1.064	1.092		0.999	1.036		0.882	1.277	*	1.006	1.075	
990	1.096	1.097		0.892	1.013	*	1.047	1.076		0.974	1.015		0.872	1.242	*	0.992	1.068	
1000	1.088	1.077		0.880	0.992	*	1.032	1.072		0.962	1.006		0.859	1.230	*	0.978	1.044	
1010	1.065	1.060		0.870	0.977	*	1.011	1.050		0.944	0.983		0.849	1.206	*	0.969	1.019	
1020	1.049	1.042		0.857	0.962	*	0.992	1.037		0.932	0.977		0.829	1.188	*	0.958	1.015	
1030	1.039	1.036		0.846	0.952	*	0.987	1.028		0.913	0.960		0.822	1.176	*	0.937	1.005	
1040	1.034	1.017		0.835	0.931	*	0.980	0.997		0.904	0.950		0.805	1.158	*	0.916	0.982	
1050	1.011	1.018		0.823	0.934	**	0.971	1.012		0.910	0.933		0.811	1.143	*	0.905	0.964	
1060	1.013	1.016		0.832	0.928	*	0.959	0.989		0.883	0.922		0.795	1.124	*	0.913	0.962	
1070	0.986	0.997		0.826	0.909		0.914	0.982		0.861	0.924		0.782	1.101	*	0.897	0.969	
1080	0.997	1.009		0.834	0.920	*	0.945	0.965		0.876	0.921		0.776	1.092	*	0.869	0.926	
1090	0.993	1.002		0.813	0.890	*	0.944	0.980		0.854	0.908		0.788	1.095	*	0.853	0.950	
1100	1.007	0.993		0.832	0.898		0.949	0.943		0.885	0.886		0.748	1.085	*	0.865	0.921	
1110	0.955	0.967		0.845	0.923	*	0.868	0.914		0.871	0.911		0.767	1.084	*	0.854	0.936	
1120	1.023	1.031		0.869	0.894		0.928	0.963		0.903	0.927		0.739	1.091	*	0.913	0.947	
1130	1.055	1.030		0.814	0.891	*	0.903	0.923		0.871	0.933		0.787	1.142	*	0.891	0.851	
1140	1.005	0.917		0.830	0.936	**	0.866	0.873		0.875	0.958		0.823	1.180	*	0.852	0.900	
1150	0.933	1.058		0.980	0.989		0.854	0.942		0.965	0.940		0.827	1.151	*	0.874	1.018	
1160	1.148	0.981		0.991	1.145	*	0.646	1.022	**	1.001	0.846	**	0.819	1.195	**	0.812	0.922	*
1170	1.178	1.519		1.681	1.097		0.873	0.956		0.965	1.028		0.898	1.109		1.024	1.027	
